# The evolutionarily conserved long non‐coding RNA *LINC00261* drives neuroendocrine prostate cancer proliferation and metastasis *via* distinct nuclear and cytoplasmic mechanisms

**DOI:** 10.1002/1878-0261.12954

**Published:** 2021-04-26

**Authors:** Rebecca L. Mather, Abhijit Parolia, Sandra E. Carson, Erik Venalainen, David Roig‐Carles, Mustapha Jaber, Shih‐Chun Chu, Ilaria Alborelli, Rebecca Wu, Dong Lin, Noushin Nabavi, Elena Jachetti, Mario P. Colombo, Hui Xue, Perla Pucci, Xinpei Ci, Cheryl Hawkes, Yinglei Li, Hardev Pandha, Igor Ulitsky, Crystal Marconett, Luca Quagliata, Wei Jiang, Ignacio Romero, Yuzhuo Wang, Francesco Crea

**Affiliations:** ^1^ Cancer Research Group‐School of Life Health and Chemical Sciences The Open University Milton Keynes UK; ^2^ Michigan Center for Translational Pathology Department of Pathology University of Michigan Ann Arbor MI USA; ^3^ Experimental Therapeutics BC Cancer Research Centre Vancouver Canada; ^4^ Institute of Pathology University Hospital Basel Switzerland; ^5^ The Vancouver Prostate Centre Vancouver General Hospital Vancouver Canada; ^6^ Department of Urologic Sciences University of British Columbia Vancouver Canada; ^7^ Molecular Immunology Unit Department of Research Fondazione IRCCS Istituto Nazionale Tumori Milano Italy; ^8^ Medical Research Institute School of Medicine Wuhan University Wuhan China; ^9^ Department of Clinical and Experimental Medicine Faculty of Health and Medical Science University of Surrey Guildford UK; ^10^ Department of Biological Regulation Weizmann Institute of Science Rehovot Israel; ^11^ Departments of Surgery, Biochemistry and Molecular Medicine Norris Cancer Center Keck School of Medicine University of Southern California Los Angeles CA USA

**Keywords:** CBX2, FOXA2, *LINC00261*, long noncoding RNA, neuroendocrine prostate cancer

## Abstract

Metastatic neuroendocrine prostate cancer (NEPC) is a highly aggressive disease, whose incidence is rising. Long noncoding RNAs (lncRNAs) represent a large family of disease‐ and tissue‐specific transcripts, most of which are still functionally uncharacterized. Thus, we set out to identify the highly conserved lncRNAs that play a central role in NEPC pathogenesis. To this end, we performed transcriptomic analyses of donor‐matched patient‐derived xenograft models (PDXs) with immunohistologic features of prostate adenocarcinoma (AR^+^/PSA^+^) or NEPC (AR^−^/SYN^+^/CHGA^+^) and through differential expression analyses identified lncRNAs that were upregulated upon neuroendocrine transdifferentiation. These genes were prioritized for functional assessment based on the level of conservation in vertebrates. Here, *LINC00261* emerged as the top gene with over 3229‐fold upregulation in NEPC. Consistently, *LINC00261* expression was significantly upregulated in NEPC specimens in multiple patient cohorts. Knockdown of *LINC00261* in PC‐3 cells dramatically attenuated its proliferative and metastatic abilities, which are explained by parallel downregulation of *CBX2* and *FOXA2* through distinct molecular mechanisms. In the cell cytoplasm, *LINC00261* binds to and sequesters *miR‐8485* from targeting the *CBX2* mRNA, while inside the nucleus, *LINC00261* functions as a transcriptional scaffold to induce SMAD‐driven expression of the FOXA2 gene. For the first time, these results demonstrate hyperactivation of the *LINC00261‐*CBX2‐FOXA2 axes in NEPC to drive proliferation and metastasis, and that *LINC00261* may be utilized as a therapeutic target and a biomarker for this incurable disease.

## Graphical abstract

Neuroendocrine prostate cancer (NEPC) is a highly aggressive disease with high incidence of metastasis. Here, we identified *LINC00261* among the top upregulated transcripts in NEPC. Inside the nucleus, *LINC00261* promoted anchorage‐independent growth and metastatic gene programs by recruiting the SMAD2/3 transcriptional machinery to *FOXA2* *cis*‐regulatory elements. Cytoplasmic *LINC00261* blocked the binding of *miR‐8485* to *CBX2* and induced a CBX2‐driven proliferative gene program. Our study demonstrates the role of *LINC00261*‐CBX2‐FOXA2 axis in proliferation and metastasis of NEPC.

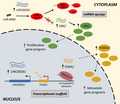

**Abbreviations**
AR: androgen receptor
CBX2: chromobox 2
CHGA: chromogranin A
ChIP‐seq: ChIP followed by massive parallel sequencing
CRPC: castration‐resistant prostate cancer
FOXA2: forkhead box A2
LINC00261: long intergenic nonprotein coding RNA 261
LincRNA: long intergenic noncoding RNA
lncRNA: long noncoding RNA
miRNA: micro‐RNA
NEPC: neuroendocrine prostate cancer
NOD/SCID: nonobese diabetic/severe combined immunodeficiency
ORF: open reading frame
PCa: prostate cancer
PDX: patient‐derived xenograft
PSA: prostate‐specific antigen
qPCR: quantitative PCR
RIP: RNA immunoprecipitation
RNA‐seq: RNA sequencing
siRNA: small‐interference RNA
SYN: synaptophysin

## 1 Introduction

Prostate cancer (PCa) is the most common male noncutaneous malignancy in the world, accounting for more than 160 000 cases a year [1]. Metastatic PCa accounts for around 6% of prostatic neoplasms at diagnosis and is associated with worse prognosis [2]. Most metastatic PCa patients are diagnosed with androgen‐dependent (AD) disease, which is currently treated with androgen‐deprivation therapy (ADT) [3]. Despite treatment, 20–30% of patients will experience progressive disease by developing castration‐resistant PCa (CRPC‐Adeno). Mechanisms involved in this process include the genetic amplification, or a gain‐of‐function mutation of the androgen receptor (AR), as well as activation of alternative signaling pathways, most notably PI3K/AKT [4]. Patients with CRPC‐Adeno are treated with second‐line hormonal and chemotherapeutic regimens [5]. Unfortunately, these second‐line interventions only postpone further disease progression. It has been reported that up to 50% of CRPCs carry hallmarks of highly aggressive neuroendocrine PCa (NEPC) [6]. This phenotype may arise from the transdifferentiation of neoplastic cells exposed to hormonal therapy [7] and can be defined by the expression of neuroendocrine markers such as chromogranin A (CHGA), neural‐specific enolase, synaptophysin, and neural cell adhesion molecule‐1.

Currently, NEPC therapy is limited to platinum‐based compounds, which may initially shrink the tumor, but do not significantly increase survival [8]. NEPC poses several therapeutic challenges due to a lack of AR expression, frequent metastatic events (particularly in visceral locations), and late diagnosis—there are currently no effective biomarkers as NEPCs do not secrete prostate‐specific antigen (PSA). In 2017, the median survival time for a patient diagnosed with metastatic NEPC was 7 months [9]. Unfortunately, the incidence of this disease is likely to rise due to the widespread use of second‐generation anti‐androgen therapeutics [4, 10]; therefore, effective therapies are urgently needed to target these cells.

Historically, cancer research has largely focused on changes in protein expression and alterations in protein‐coding regions, which comprise just 2% of the human genome. A major portion of the genome comprises lncRNAs, which are defined as noncoding transcripts longer than 200 bp. There are approximately 60 000 lncRNAs in the human genome [11], most of which are functionally uncharacterized. lncRNAs are often expressed at very low levels in human cells, but some of these transcripts are more abundant and regulate important pathophysiological functions [12]. While most lncRNAs are species‐specific, a small fraction of these transcripts is highly conserved and is thought to regulate key cellular functions [13]. Some evolutionarily conserved lncRNAs have been identified as master regulators of cancer progression [13]. Here, we describe for the first time a highly conserved lncRNA, long intergenic nonprotein coding RNA 261 (*LINC00261*; aka *DEANR1; NC_000020.11*), as a key driver of NEPC proliferation and metastasis, respectively, through regulation of chromobox 2 (CBX2) and forkhead box A2 (FOXA2) expression. We delineate two distinct mechanisms through which *LINC00261* either functions as a transcriptional scaffold for the FOXA2 gene in the nucleus or a micro‐RNA (miRNA) sponge for the *CBX2* transcript in the cytoplasm.

## 2 Materials and methods

### 2.1 RNA‐seq analysis of PDX models

RNA samples were isolated from patient‐derived xenograft models (PDXs) as previously described by Ref. [14]. RNA sequencing (RNA‐seq) and bioinformatic analysis were carried out by the Centre for Genomics, University of Liverpool. The resulting dataset was further analyzed to determine lncRNAs differentially expressed between hormone‐sensitive prostatic adenocarcinoma (LTL‐331) and NEPC (LTL‐331R). Thresholds for expression were set as log_2_ fold change minimum −4; false discovery rate < 0.1; and fragments per kilobase of transcript per million mapped reads (FPKM) greater than 10. Coding potential was assessed using NCBI's nucleotide BLAST (blast.ncbi.nlm.nih.gov), Ensembl (http://www.ensembl.org), Coding Potential Calculator (http://cpc.cbi.pku.edu.cn), and TestCode software (http://www.bioinformatics.org/sms/testcode.html). The shortlist was then further refined such that transcripts were ranked based on evolutionary conservation using methods described in Ref. [15].

### 2.2 RNA‐seq analysis of *LINC00261* knockdown in PC‐3 cells

RNA samples were isolated from PC‐3 cells cultured and treated with 10 nm small‐interference RNAs (siRNAs) as described in Materials and methods for siRNA reverse transfection. RNA‐seq and bioinformatic analysis were carried out by Molecular Pathology Unit, Universitätsspital Basel, Switzerland. The resulting dataset was further analyzed to determine differentially expressed transcripts between negative control siRNA (NC) and *LINC00261* siRNA (siRNA 1). Thresholds for expression were set as log_2_ fold change above 2 (upregulated) and below −2 (downregulated).

### 2.3 cBioPortal analysis

Shortlisted lncRNAs were queried using cBioPortal (http://www.cbioportal.org) to assess clinical relevance and co‐expression analysis. The datasets used for these analyses are described in relevant figure legends. Data were obtained from publicly available RNA‐seq datasets and sorted by type of prostatic neoplasm [hormone‐naive (HN), CRPC, or NEPC].

In order to analyze copy number variations in NEPC‐associated pathways, *LINC00261* and AKT signaling pathway genes were queried. 17 AKT pathway‐associated genes were queried using cBioPortal's inbuilt ‘general PI3K‐AKT‐mTOR signaling’ query. This included *PIK3CA, PIK3R1, PIK3R2, PTEN, PDPK1, AKT1, AKT2, FOXO1, FOXO3, MTOR, RICTOR, TSC1, TSC2, RHEB, AKT1S1, RPTOR*, and *MLST8*. The dataset used for copy number analysis was from the referenced study [16].

### 2.4 TANRIC analysis

The TANRIC analysis was carried out on shortlisted lncRNAs (https://ibl.mdanderson.org/tanric/_design/basic/query.html) to assess survival and to determine associated mRNAs. lncRNAs were queried based on their alias shown in Table S1, or by Ensembl ID, all datasets were analyzed.

### 2.5 GEO profile data

Expression of *LINC00261* in a panel of cell lines was obtained from the NCI‐60 cancer cell line panel (GEO ID 86803759). Data were downloaded from https://www.ncbi.nlm.nih.gov/geo/tools/profileGraph.cgi?ID=GDS4296:228004_at and visualized using graphpad prism 7 software (https://www.graphpad.com/scientific‐software/prism/).

### 2.6 LTL microarray analysis

The Living Tumour Laboratory data were obtained from the gene expression database found at www.livingtumourlab.com. Samples from HN, CRPC, and NEPC tumors were used. Microarray data were downloaded from the PCa datasets and log_2_‐transformed.

### 2.7 Cell culture

RWPE‐1 cells were obtained from the American Tissue Culture Collections (ATCC, Middlesex, UK) and were cultured in keratinocyte serum‐free media (KSFM; Gibco, Loughborough, UK) supplemented with 0.05 mg·mL^−1^ bovine pituitary extract (BPE) and 5 ng·mL^−1^ human recombinant epidermal growth factor (EGF) and 1% Pen‐Strep (Gibco). LNCaP (clone FGC), PC‐3, and DU‐145 cells were obtained from ATCC and were cultured in RPMI‐1640 (Gibco) supplemented with heat‐inactivated FBS (Gibco) and 1% Pen‐Strep (Gibco) for all assays but MTT assays where phenol red‐free media was used. Murine cell lines T23 [17] and OPT7714 [18] were kindly provided by Elena Jachetti and Mario P Colombo (Istituto Nazionale Tumori, Milan, Italy) and were cultured in Dulbecco's Modified Eagle Medium (high‐glucose; Gibco), supplemented with 10% heat‐inactivated GBS (Gibco), 10 mm sodium pyruvate (Gibco), 10 mm HEPES (Gibco), 2 mm l‐glutamine (Gibco), and 1% Pen‐Strep (Gibco). All cells were cultured at 37 °C in a humidified environment containing 5% CO_2_. Cells prepared for *in vivo* work were transduced using constructs generated and packaged by Genecopoeia (Rockville, MD, USA). PC‐3 cells were transduced using media supplemented with polybrene (aka hexadimethrine bromide; Sigma, Gillingham, Dorset, UK) at a final concentration of 8 µg·mL^−1^. Cells were incubated with purified human *LINC00261* lentiviral particles overnight and were then washed with complete media. 48–72 h post‐transduction, stably transduced cells were isolated using puromycin selection (Gibco). Selection was carried out for 2 weeks at a concentration of 1 µg·mL^−1^.

For inoculation of animals, the trypsinized cells were washed 1× with PBS. The cells were counted with an automated cell counter (TC20; Bio‐Rad, Hercules, CA, USA) and resuspended at 10^7^ cells·mL^−1^ in PBS for inoculation.

### 2.8 Analysis of gene expression

RNA was isolated from cells in culture using the RNeasy Plus Mini Kit (Qiagen, Manchester, UK) according to manufacturer's instructions. Reverse transcription was carried out using 1 μg RNA per reaction using the High‐Capacity cDNA Reverse Transcription Kit (Applied Biosystems, Loughborough, UK) according to kit instructions. The resulting cDNA was diluted one in 10 for RT‐quantitative PCR (qPCR) analysis. cDNA panels of non‐neoplastic tissue were purchased from Clontech (Saint‐Germain‐en‐Laye, France). TaqMan probes were used as stated in each experiment according to the manufacturer's instruction. Human assays were obtained from Thermo Fisher (Loughborough, UK): *LINC00261* (Hs03679073_m1), *LINC00491 (NEAR3)* (Hs01374494), *LOC101929331* (*NEAR5*) (Hs04406674_m1), *LOC100507175* (*NEAR8) (Hs01388460_m1), LINC01612* (*NEAR16*) (Hs04407222_m1), *CTD‐2151A2.1* (*NEAR18*) (Hs04404647), *LINC00616* (*NEAR21*) (Hs04232282_m1), *HPRT1* (Hs02800695), *FOXA2* (Hs00232764_m1), and *CBX2* (hs01034268_m1). Murine assays were also obtained from Thermo Fisher *9030622O22‐Rik* (Mm01335331_m1), and *Hprt1* (Mm03024075_m1).

### 2.9 Localization of lncRNA

RNA was isolated from cells in culture in nuclear and cytoplasmic fractions, which were separated using the PARIS Kit (Ambion, Loughborough, UK) according to manufacturer's instruction. For validation and localization studies, RT‐qPCR was used with the probes *MALAT1* (Hs00273907_s1), *GAPDH* (Hs02786624_g1), and *HPRT1* (Hs02800695_m1).

### 2.10 siRNA reverse transfection

Knockdown studies were performed using the reverse transfection method. Cells were seeded in a six‐well or 96‐well plate with lipid:siRNA mixture prepared using Lipofectamine 2000 (Invitrogen, Loughborough, UK) as per manufacturer's protocol. Final siRNA concentrations were 10 nmol. All dicer substrate short interfering RNAs against *LINC00261* were purchased from Integrated DNA Technologies (Leuven, Belgium) [hs.Ri.LINC00261.13.1 (siRNA 1), hs.Ri.LINC00261.13.2 (siRNA 2), and hs.Ri.LINC00261.13.3 (siRNA 3)]. Control siRNAs were also purchased from IDT (negative control DS NC1 and positive control HPRT‐S1 DS). For murine cells, silencer select siRNAs against *9030622O22‐Rik* were purchased (siRNA 1 = n404958, siRNA 2 = n440961, and siRNA 3 = n518634, as well as negative control; Thermo) and transfected with 25 nm in the same manner previously described. RNA was isolated using RNeasy Plus Mini Kit (Qiagen).

### 2.11 MTT assays

Cells were plated in 96‐well plates and treated with siRNA as previously described. MTT assays were carried out by first incubating with DMSO or media for 1 h. After this 5 mg·mL^−1^, thiazolyl blue tetrazolium bromide (MTT; Sigma) was added at 10% of the well volume. The cells were incubated with MTT for 3 h. After this, all media were removed and MTT was solubilized using 50 µL DMSO. The plate was then read using a BMG OPTIMA POLARstar plate reader (BMG Labtech, Aylesbury, UK) at 562 and 680 nm. MTT assays were corroborated by manually cell counting bright‐field images of cultured cells.

### 2.12 Caspase 3/7 assays

Cells were plated in a white, flat‐bottomed 96‐well plates and treated with siRNAs as previously described. On day 3 post‐transfection, Caspase‐Glo reagent was added per well (Promega, Southampton, UK) according to manufacturer's instruction, was added to well, and incubated for 1.5 h. Luminescence was then quantified using the BMG POLARstar plate reader (BMG Labtech).

### 2.13 Wound‐Healing assay (migration)

PC‐3 cells were reverse‐transfected for 18 h with 10 nmol siRNAs 2 and 3 as these did not yield a significant reduction in viable cells as determined by MTT assays.

After 18 h, a scratch was made in the cell monolayer using a sterile P20 pipette tip. Cells were imaged periodically across the wound until the wound was closed. Images were analyzed using the MRI wound‐healing tool, ImageJ.

### 2.14 Western blot

Cell lysates were created using 100 μL of RIPA buffer [Tris pH 8.0 (Sigma); NaCl (Sigma); EDTA (Sigma); IGEPAL (Sigma); SDS (Sigma), NaF (Sigma); NaVO_3_ (Sigma)]. Protein was quantified using Pierce BCA assay according to manufacturer's protocol (Thermo). Ten microgram of protein was resolved by gel electrophoresis on reducing SDS/PAGE (Tris/glycine 4–20%, Thermo) run at 125 V for 2 h. The proteins were then transferred to nitrocellulose membrane at 350 Ma for 1.5 h. The membranes were blocked in 5% nonfat milk dissolved in Tris‐buffered saline (TBS; Sigma) at room temperature for 1 h. Following this, blots were incubated overnight at 4 °C with protein‐specific primary antibodies in 5% milk in TBS supplemented with 2% Tween‐20 (Sigma). Antibodies used were RabMab anti‐FOXA2 (Ab108422; Abcam, Cambridge, UK) and RabMab anti‐HPRT1 (ab133242, Abcam). After incubation, blots were washed five times in TBS‐T for 10 min each. Lastly, blots were incubated with HRP‐conjugated anti‐mouse secondary antibody diluted in 10 mL of TBS‐T at room temperature for 1 h [goat anti‐rabbit IgG (Sigma)]. After washing as above, ECL Western Blotting Substrate Kit was used (Millipore, Watford, UK) and blots were visualized using Syngene Gbox with genetools software (Syngene, Bangalore, India).

### 2.15 Clinical sample analysis (FFPE RNA isolation)

Patient tissue was obtained in the form of FFPE blocks from the Royal Surrey County Hospital, in Guildford, UK. All patients had histologically proven PCa and had failed at least three lines of conventional therapies. Patients consented to the evaluation of their tissue, and their study was approved by the local ethics committee ref. 12/LO/1661. NEPC samples were defined histologically as having at least moderate expression of one of the following markers SYP, ENO2, and CHGA. AD samples were defined histologically and having expression of AR and/or PSA, and being negative for NE markers. FFPE blocks were obtained from the University of Surrey. RNA was isolated from the FFPE blocks using the RecoverAll Extraction Kit (Thermo). For each sample, an average of 800 ng of total RNA was reverse‐transcribed using the SuperScript VILO cDNA Synthesis Kit (Thermo). RNA isolation and cDNA synthesis were performed in the Contract R&D Unit of the Institute of Pathology and Medical Genetics of Basel. The resulting cDNA was used for qPCR analysis of *LINC00261* and *HPRT1* using the TaqMan probes described previously.

### 2.16 RNA immunoprecipitation assay

An RNA‐binding protein immunoprecipitation (RIP) assay was performed using the Magna RIP Kit (Millipore) according to manufacturer's instruction. Cell lysates from 10 × 10^6^ cells and 2–5 µg of control IgG or antibody against SMAD2/3 (R&D Systems; AF3797, Abingdon, UK) were used. We validated the RIP assay using the SNRNP70 antibody, which can bind to U1 snRNA. A ChIP assay was performed using magnetic beads. Equal amounts of chromatin for each sample and 1 µg of control IgG or antibody against SMAD2/3 were used.

### 2.17 Animal study

Immunocompromised mice (NOD.Cg‐Rag1^tm1Mom^ Il2rg^tm1Wjl^/SzJ) were purchased from the Animal Resources Centre at BC Cancer Research Centre. All procedures performed were approved by the Animal Care Committee at the University of British Columbia (Certificate # A16‐0304). Six‐week‐old mice were inoculated with PC‐3‐NC (nontargeting shRNA knockdown) or shLINC00261 (*LINC00261*KD) cells at both left and right flanks. Each flank was inoculated with 10^6^ viable cancer cells, accurately counted using the trypan blue counter viability stain. Four mice were inoculated with each cell line; therefore, there were eight inoculation sites per cell line. The mice were monitored twice per week for palpable tumor and/or tumor measurement, and the monitoring was started 1 week after cell inoculation. Dimensions of tumors were measured with a caliper, and the tumor volume was calculated with the formula: tumor volume (mm^3^) = 0.523 × length (mm) × width^2^ (mm^2^).

### 2.18 Zebrafish embryo metastasis assessment

Wild‐type ABTL zebrafish were maintained in aquaria according to standard protocols. Embryos were generated by natural pairwise mating and raised at 28.5 °C on a 14‐h light/10‐h dark cycle in a 100‐mm Petri dish containing aquarium water with methylene blue to prevent fungal growth. All experiments were performed with 2‐ to 7‐day‐old embryos postfertilization and were done in approved University of Michigan fish facilities using protocols approved by the University of Michigan Institutional Animal Care and Use Committee (UM‐IACUC). Cell injections were carried out as previously described [19]. In brief, GFP‐expressing NC‐treated, si *LINC00261*‐treated, or siFOXA2‐treated PC‐3 cells were resuspended in PBS at the concentration of 1 × 10^7^ cells·mL^−1^. Forty‐eight hours after fertilization, wild‐type embryos were dechorionated and anesthetized with 0.04 mg·mL^−1^ tricaine. Approximately 10 nL (approximately 100 cancer cells) was microinjected into the perivitelline space using a borosilliac micropipette tip with filament. Embryos were returned to aquarium water and washed twice to remove tricaine, and then moved to a 96‐well plate with one embryo per well and kept at 35 °C for the duration of the experiment. All embryos were imaged at 24‐h intervals to follow metastatic dissemination of injection cells. Water was changed daily to fresh aquarium water. More than 30 fish were injected for each condition (wild‐type no. 2, *n* = 30; wild‐type no. 5, *n* = 50; wild‐type no. 57, *n* = 35; wild‐type no. 84, *n* = 57; wild‐type no. 113, *n* = 38), and metastasis was visually assessed daily up to 5 days after injection (i.e., for a total of 7 days postfertilization) by counting the total number of distinct cellular foci in the body of the embryos. All of the metastasis studies were terminated at 7 days postfertilization in accordance with the approved embryo protocols. Embryos were either imaged directly in the 96‐well plates or placed onto a concave glass slide to capture representative images using a fluorescent microscope (Olympus IX71). For quantification, evidently distinct cell foci in the embryo body were counted 72 h after the injections.

### 2.19 miR mimic and inhibitor studies

PC‐3 cells were reverse‐transfected with 30 nm NC or miR‐8485 inhibitor/mimic using the method previously described. The sequences used were obtained from Thermo Fisher and were as follows: miR‐8485 inhibitor (NH31809); miR‐8485 mimic (MC31809); NC inhibitor (4464076); and NC mimic (4464058).

### 2.20 Analysis of miR expression

For miRNA analysis, RNA was isolated using miRNeasy (Qiagen, Manchester, UK) according to manufacturer's instructions. Reverse transcription was carried out using the miRNA cDNA Synthesis Kit (Applied Biosystems, Loughborough, UK) according to kit instructions using the reverse transcription primers provided with miR‐8485 (480631_mir), or U6 snRNA (001973) using 10 ng RNA per reaction. The resulting cDNA was diluted 1 in 10 for RT‐qPCR analysis using the qPCR TaqMan probes provided in the kits described for miR‐8485 or U6 according to manufacturer's instructions.

### 2.21 miRNA‐binding dual‐luciferase reporter assay

For the miRNA‐binding assay, a 100‐bp segment centered at the miR‐8485 seed sequence (5′‐GTATGTGTGTGCGTGTGTGTTT‐3′) in 3′UTR of *LINC00261* was cloned into the firefly luciferase gene using the pmirGLO dual‐reporter system (Promega; E1330). For the control vector, the same *LINC00261* segment with the seed sequence replaced with a random sequence (5′‐GTATGTCCATGAAGCCATTGTC‐3′) was cloned into the luciferase gene. Cloning was performed using the NheI‐HF (NEB; R3131L) and AccI (NEB; R0161S) restriction enzymes following the manufacturer's instructions. Cloned sequences were verified through the Sanger sequencing. A total of 4 × 10^6^ HEK293FT cells were seeded in 10‐cm dishes and transfected the next day with 10 μg of either unmodified pmirGLO vector or vectors encoding the *LINC 00261* or the mutant*‐LINC00261* sequences. After 10 h, the transfected cells were plated into Poly‐d‐lysine 12‐well plates at 400 000 cells per well and transfected with 25 or 50 nm of mirVana miRNA mimic negative control #1 (Thermo Fisher Scientific; 4464058) or has‐miR‐8485 (Thermo Fisher Scientific; Cat. No. 4464066; miRBase Accession No. MIMAT0033692). After 48 h, cells were lysed using 100 μL of passive lysis buffer for 5 min at room temperature followed by two rounds of freeze–thaw cycles and were centrifuged at 4 °C for 10 min at max speed (> 10 000 ***g***). The supernatant was then diluted 1/5 or 1/10, and dual‐luciferase activity was recorded as per manufacturer's protocol (Promega; E2920) using the TECAN Infinite M1000 Pro Plate Reader, with each condition having 3–6 independent replicates. The plate reader was set to automatic attenuation with 1000‐ms integration time. Cell number variation and/or differences in transfection efficiencies were controlled by normalizing the firefly luciferase signals with the matched Renilla luciferase signals. The firefly‐to‐Renilla ratios values were then normalized using signals from the unmodified pmirGLO‐treated cells to control for any off‐target binding of the miR‐8485 to the luciferase transcripts.

### 2.22 Immunohistochemistry

Formalin‐fixed, paraffin‐embedded tissue sections were prepared, and histopathologic and immunohistochemical analyses were performed as previously described [20]. IHC staining was carried out using anti‐Ki67 (Thermo Fisher, RM‐9106, 1 : 100). Biotinylated secondary antibody (Vector Laboratories) peroxidase‐linked avidin/biotin complex reagents (Vector Laboratories) and diaminobenzidine (DAB, Sigma‐Aldrich) were used for the staining. Sections were imaged using 10X objective of Microphot‐fx (Nikon) using Surveyor software (Objective Imaging). The percent staining per section of Ki67 was evaluated using imagej by setting a threshold and analyzing particles. Five random areas were analyzed per section.

### 2.23 Overexpression of *LINC00261* in DU‐145 cells

*LINC00261* open reading frame‐encoding plasmids were kindly provided by the laboratory of Dr Crystal Marconett and were previously described [21]. DU‐145 cells were maintained as previously described before being transfected with either an empty vector or *LINC00261* plasmids using Lipofectamine 3000 (Thermo Fisher) using 2.5 µg DNA for 72 h according to manufacturer's instructions.

### 2.24 *In silico* prediction of miRNA target genes

*In silico* predictions for miRNA‐lncRNA‐binding site were carried out with the online open‐access lncBase v.2 database (http://carolina.imis.athena‐innovation.gr/diana_tools/web/index.php?r=site%2Ftools). Predicted miRNAs are ranked based on probability of their binding in a previously published DIANA‐LncBase v2 database [22]. LncBase v.2 scores predicted miRNA targets based on preset parameters and assigned a score to the predicted miRNA and used it to rank them on the screen. The table shown in the figure are the different types of bindings predicted by the database. Top‐ranked miRNA was selected for further study. Prediction for miRNA–mRNA interactions was investigated using four different independent online, open‐access databases (TargetScan, RNA22, mirDB, and miRWalk). Predicted target mRNAs were overlapped with genes downregulated upon LINC00261 knockdown (RNA‐seq) in PC‐3 cells. Top ranking mRNAs were prioritized for functional validation. Direct links to the databases are included below:

TargetScan (http://www.targetscan.org/vert_72/); RNA22 (https://cm.jefferson.edu/rna22/); mirDB (http://mirdb.org/); and miRWalk (http://mirwalk.umm.uni‐heidelberg.de/).

### 2.25 ChIP‐seq and data analysis

ChIP experiments were carried out using the HighCell# ChIP‐Protein G Kit (Diagenode) as per the manufacturer's protocol. Chromatin from five million cells was used per ChIP reaction with 10 μg of the SMAD2/3 protein antibody (Abcam: ab202445). In brief, cells were trypsinized and washed twice with 1× PBS, followed by crosslinking for 8 min in 1% formaldehyde solution. Crosslinking was terminated by the addition of 1/10 volume 1.25 m glycine for 5 min at room temperature followed by cell lysis and sonication (Bioruptor, Diagenode), resulting in an average chromatin fragment size of 200 bp. Fragmented chromatin was then used for immunoprecipitation using various antibodies, with overnight incubation at 4 °C. ChIP DNA was de‐crosslinked and purified using the iPure Kit V2 (Diagenode) using the standard protocol. Purified DNA was then prepared for sequencing as per the manufacturer's instructions (Illumina). ChIP samples (1–10 ng) were converted to blunt‐ended fragments using T4 DNA polymerase, *Escherichia coli* DNA polymerase I large fragment (Klenow polymerase), and T4 polynucleotide kinase [New England BioLabs (NEB)]. A single A base was added to fragment ends by Klenow fragment (3′–5′ exo minus; NEB) followed by ligation of Illumina adaptors (Quick ligase; NEB). The adaptor‐ligated DNA fragments were enriched by PCR using the Illumina barcode primers and Phusion DNA polymerase (NEB). PCR products were size‐selected using 3% NuSieve Agarose Gels (Lonza) followed by gel extraction using QIAEX II reagents (Qiagen). Libraries were quantified and quality‐checked using the Bioanalyzer 2100 (Agilent) and sequenced on the Illumina HiSeq 2500 Sequencer (125‐nucleotide read length).

Paired‐end, 125‐bp reads were trimmed and aligned to the human reference genome (GRC h38/hg38) with the Burrows–Wheeler Aligner (BWA 0.7.17‐r1198). The SAM file obtained after alignment was converted into BAM format using samtools (Version: 1.9). MACS2 callpeak was used for performing peaking calling with the following option: ‘macs2 callpeak–call‐summits–verbose 3 ‐g hs ‐f BAM ‐n OUT–qvalue 0.05’. For H3K27ac data—broad option was used. Using deepTools bamCoverage, a coverage file (bigWig format) for each sample was created. The coverage is calculated as the number of reads per bin, where bins are short consecutive counting windows. While creating the coverage file, the data were normalized with respect to each library size. ChIP peak profile plots and read‐density heat maps were generated using deepTools, and cistrome analyses were carried out using the ChIPpeakAnno or ChIPseeker packages in r.

### 2.26 Soft agar colony formation assay

PC‐3 cells were first treated with either 50 nm of nontargeting control siRNA or siRNA targeting the LINC00261 or FOXA2 transcripts using the RNAiMAX reagent as described above. Twenty‐four hours after siRNA transfections, 5000 or 10 000 viable PC‐3 cells were layered in 1 mL of 0.3% Noble agar (Sigma‐Aldrich; CAS Number 9002‐18‐0) over an already solidified layer of 0.6% Noble agar per well of a six‐well plate, with 200 μL of complete media on the top to prevent desiccation. The cells were then allowed to form colonies for 14 days with media being replenished every 3 days, after which they were stained with iodonitrotetrazolium chloride (Sigma‐Aldrich; CAS Number 146‐68‐9) and imaged using a scanner. Distinct individual colonies were manually counted from three randomly chosen 10× fields per well and averaged. This assay was carried out at least two times with two replicates per condition per experiment.

### 2.27 Ethical approval statements

FFPE samples were obtained from the University of Surrey. Patients consented to the evaluation of their tissue, and their study was approved by the local ethics committee reference 12/LO/1661. All murine procedures performed were approved by the Animal Care Committee at the University of British Columbia (Certificate # A16‐0304). Animals were housed in a certified animal facility at the British Columbia Cancer Research Centre—Animal Resource Centre (Vancouver, Canada) following all ethical housing procedures and daily weekday monitoring. PDXs were maintained by transplantation into subrenal capsules of male nonobese diabetic/severe combined immunodeficiency (NOD‐SCID) mice as previously described (Lin *et al*. [20]). All animal experiments were performed following the ethical guidelines and standards set by the Declaration of Helsinki and in accordance with the established animal care and use protocols approved by the UBC Animal Care Committee (protocol #: A10‐0100). All zebrafish procedures were carried out in approved University of Michigan fish facilities using protocols approved by the University of Michigan Institutional Animal Care and Use Committee (UM‐IACUC).

## 3 Results

**Fig. 1:**
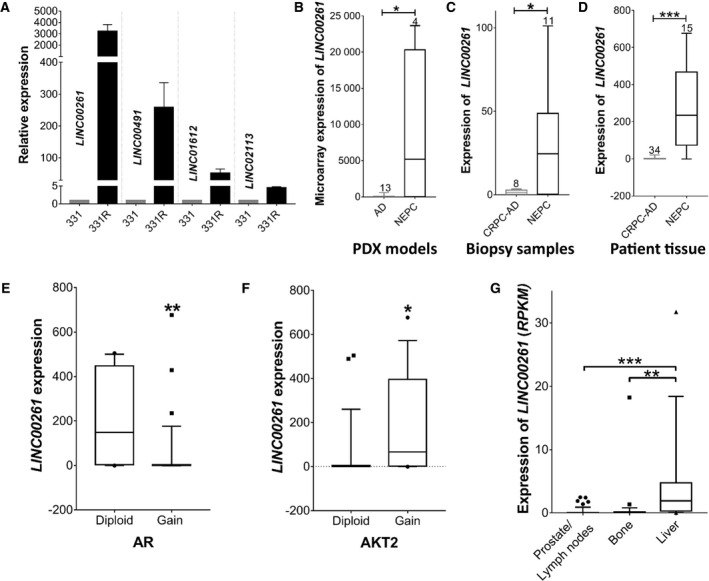
*LINC00261* is associated with NEPC. (A) Expression of (TaqMan qPCR) of select *lincRNAs* that are upregulated in NEPC in the LTL‐331/331R PDX model. (B) Relative expression (microarray) of *LINC00261* in several PDX models of prostate adenocarcinoma (*n* = 13) and NEPC (*n* = 4) from the Living Tumor Laboratory. (C) Relative expression (qPCR) of *LINC00261* in FFPE biopsy samples from NEPC (*n* = 11) and CRPC‐Adeno (*n* = 8) patients. (D) Expression of *LINC00261* (RNA‐seq) in patient specimens with features of neuroendocrine (*n* = 15) or castration‐resistant (*n* = 34) disease. Datasets used is from Ref. (Barbieri *et al*. [54]; Beltran *et al*. [16]) (E) *LINC00261* expression in patient samples with or without AR copy gains (**P = 0.0025), or (F) AKT2 copy gains, an activating component of the PI3K/AKT pathway (*P = 0.0197). (G) *LINC00261* expression in clinical PCa biopsies from secondary lesions in the prostate/lymph nodes (*n* = 25), bone (*n* = 29), or liver (*n* = 17; ***P < 0.001, ANOVA and Tukey's *post hoc* test). Data from cBioPortal, Metastatic PCa SU2C/PCF Dream Team Cell, 2015. Data in B–E were analyzed by two‐tailed unpaired *t*‐test. All data are presented as mean ± SEM with at least two independent biological replicates.

Using previously described high‐fidelity patient‐derived xenograft (PDX) models [20], we sought to identify lncRNAs that could be functionally implicated in NEPC pathogenesis. Using RNA‐seq, we profiled transcriptomes of a well‐studied, donor‐matched PDX pair that show divergent histopathologic features: LTL‐331 (resembles prostate adenocarcinoma) and LTL‐331R (resembles NEPC). Differential expression analyses between LTL‐331R vs LTL‐331 revealed that out of the 15 224 identified lncRNAs, only 32 genes were upregulated in the neuroendocrine model that met our stringent criteria of log_2_ fold change ≥ 4, FDR < 0.01, and FPKM > 10. Second‐pass filtering using Coding Potential Calculator (http://cpc.cbi.pku.edu.cn/) and TestCode (https://www.bioinformatics.org/sms/testcode.html) software yielded the final list of 26 *bona fide* lncRNAs, which were then ranked by level of conservation depending on the number of orthologs in 17 species (16 vertebrates and one invertebrate) as previously described [15] (Fig. S1A). Here, *LINC00261* emerged as the most conserved gene with marked upregulation in the neuroendocrine disease (Fig. S1B). Notably, using TaqMan qPCR we were able to confirm over 3229‐fold overexpression of *LINC00261* in LTL‐331R vs LTL‐331 (Fig. 1A), and additionally validated three other nominated transcripts for which a predesigned TaqMan probe was available.

### 3.1 *LINC00261* is selectively upregulated in human specimens of NEPC

To assess whether our RNA‐seq results could be generalized, we investigated the expression of *LINC00261* in other biologically independent PDX models from the Living Tumour Laboratory (http://www.livingtumorlab.com/). We found *LINC00261* to be significantly upregulated in four NEPC PDX tumor lines compared with 13 hormone‐naïve tumor lines (*P* = 0.0102; Fig. 1B). These results were further corroborated using an in‐laboratory patient biopsy cohort comprising of 8 adenocarcinomas and 11 NEPC formalin‐fixed specimens, where *LINC00261* expression (qPCR) was elevated in the neuroendocrine samples (*P* = 0.0387; Fig. 1C).

Next, we investigated whether *LINC00261* expression could distinguish between CRPC‐Adeno and NEPC using a publically available patient database [16], which includes genomic and transcriptomic data from 34 CRPC and 15 NEPC tumors. Again, *LINC00261* had a significantly higher expression in NEPC patients by 161‐fold compared with CRPC‐Adeno patients (*P* = 0.0003; Fig. 1D). Using the same clinical dataset, we explored whether *LINC00261* is associated with other markers of neuroendocrine disease (Table S1). Our analysis revealed a very significant correlation between *LINC00261* and *CHGA, ENO2, CBX5*, and *NCAM‐1* (Table S1). Notably, *LINC00261* expression was negatively correlated with *AR* amplification (*P* = 0.0025; Fig. 1E), but positively correlated with *AKT2* amplification (*P* = 0.0197; Fig. 1F). *LINC00261* expression was also significantly upregulated in metastatic PCa lesions from the liver as opposed to other sites (Fig. 1G), which often show neuroendocrine features [23]. Taken together, these results show that *LINC00261* is selectively upregulated in human NEPC relative to both hormone‐sensitive and hormone‐independent adenocarcinomas, where its expression is positively associated with classical neuroendocrine markers.

### 3.2 *LINC00261* is required for survival and invasiveness of NEPC

**Fig. 2:**
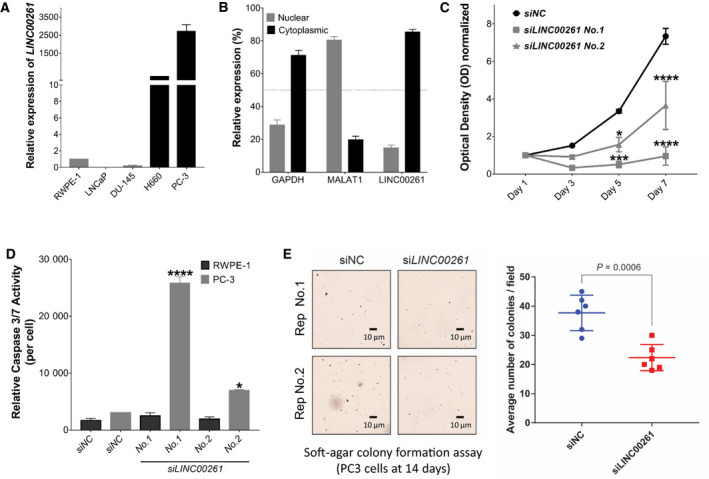
*LINC00261* is required for NEPC cell viability and invasiveness. (A) Expression (qPCR) of *LINC00261* in a panel of prostate cell lines. (B) Subcellular localization of *LINC00261, GAPDH,* and *MALAT1* RNA transcripts in PC‐3 cells. (C) Viability (MTT assay) of PC‐3 cells treated with a control, nontargeting siRNA (siNC), or distinct siRNAs targeting *LINC00261* at 10 nm dosage. (D) Cell viability‐adjusted activity of caspase 3/7 after 72 h of siRNA‐mediated *LINC00261* knockdown in PC‐3 cells. (E) Left, Representative images of iodonitrotetrazolium chloride stained soft agar PC‐3 cell colonies after treatment with either the nontargeting, control siRNA (siNC), or siRNA targeting the *LINC00261* (si*LINC00261*) transcript (two‐tailed *t*‐test). Right, Average number of soft agar colonies per 10× field of siNC‐ or si*LINC00261*‐treated PC‐3 cells. The scale bar at the bottom marks a 10 μm width. Distinct cells colonies were counted from three randomly chosen 10X field per well from two biological replicates. All data are presented as mean ± SEM with at least two independent biological replicates. (C) Analyzed using two‐way ANOVA with Tukey's multiple comparisons test. (D) Analyzed by one‐way ANOVA with Dunnett's *post hoc* test. (E) Analyzed by two‐tailed *t*‐test. *P < 0.05; ****P < 0.001.

First, using qPCR we profiled the expression of *LINC00261* in a panel of human prostate cell lines: RWPE‐1 (non‐neoplastic); LNCaP (AR mutant, hormone‐sensitive PCa); DU‐145 (AR‐negative); H660 and PC‐3 (NEPC models that are AR‐negative and express NE markers [7, 24]). Consistent with the clinical findings, *LINC00261* was highly upregulated in the neuroendocrine H660 (227‐fold vs RWPE‐1) and PC‐3 cells (2714‐fold vs. RWPE‐1, Fig. 2A), compared with both normal prostate and AR‐positive PCa cells. Next, subcellular fractionation was followed by qPCR, using a nuclear‐retained lncRNA (*MALAT1*) and a mRNA (*GAPDH*) as controls, as previously described [25, 26, 27]. This experiment revealed roughly 80% of the transcript to be cytoplasmic, while the rest localized within the nucleus (Fig. 2B). Given the high conservation of *LINC00261*, we also profiled the expression of its murine ortholog *9030622O22‐Rik* in cell lines derived from murine PCa [17, 18]. Our results showed a dramatic upregulation of *9030622O22‐Rik* in OPT7714 cells (NEPC) by 120‐fold compared with T23 cells (adenocarcinoma; Fig. S2A); and in OPT7714, the transcript was equally abundant in the cytoplasmic and nuclear compartments (Fig. S2B) This suggests that both expression and cellular localization of *LINC00261* are conserved between human and murine NEPC cells.

Given the highest expression of *LINC00261* in PC‐3 cells, we selected this cell line for the functional studies. We first validated three distinct siRNAs targeting *LINC00261*. With only 10 nm treatment of individual siRNAs, we achieved 50–75% knockdown efficiency within 48h after transfection relative to the control, nontargeting siRNA‐treated cells (Fig. S2C). Notably, knockdown of *LINC00261* in PC‐3 cells dramatically attenuated their proliferative ability starting from day 3 (Fig. 2C). At this time point, under the microscope, *LINC00261*‐silenced cells showed evident morphological signs of apoptosis (cell shrinkage, membrane blebbing, etc.; Fig. S2D) and reduced number of viable cells (Fig. S2E), which we confirmed using the caspase 3/7 activity assay. *LINC00261* knockdown with two distinct siRNAs in PC‐3 cells resulted in significant activation of the executioner caspases 3/7 that irreversibly commits the cells to death *via* apoptosis (*siL*INC*00261* No. 1 *P* = 0.0001; *siLINC00261* No. 2 *P* = 0.0036; Fig. 2D). Notably, knockdown of *LINC00261* in non‐neoplastic RWPE‐1 cells did not significantly affect cell growth or induce caspase 3/7 activation (Fig. 2D). Conversely, overexpression of *LINC00261* in DU‐145 cells induced a significant increase in cellular growth, compared with cells expressing the empty control vector (*P* = 0.003; Fig. S2F).

Since NEPC is characterized by an elevated metastatic rate, we decided to investigate the effects of *LINC00261* silencing on migration and anchorage‐independent growth. We transiently treated PC‐3 cells with distinct siRNAs for 18h and assessed their migratory abilities using the scratch wound‐healing assay or their invasive abilities using the Boyden chamber assay. Importantly, within 18h we were able to achieve greater than 60% target knockdown (Fig. S3A) and had no impact on cell viability (Fig. S3B). While we found no changes in the migration ability (Fig. S3C,D), *LINC00261* knockdown significantly attenuated the ability of PC‐3 cells to form colonies in soft agar relative to the control cells (*P* = 0.0006; Fig. 2E). Taken together, these results demonstrate that *LINC00261* is required to maintain the hyperproliferative and anchorage‐independent survival of NEPC cells, which are two defining pathologic features of this disease.

### 3.3 *LINC00261* is required for the proliferation of NEPC cells *in vivo*

**Fig. 3:**
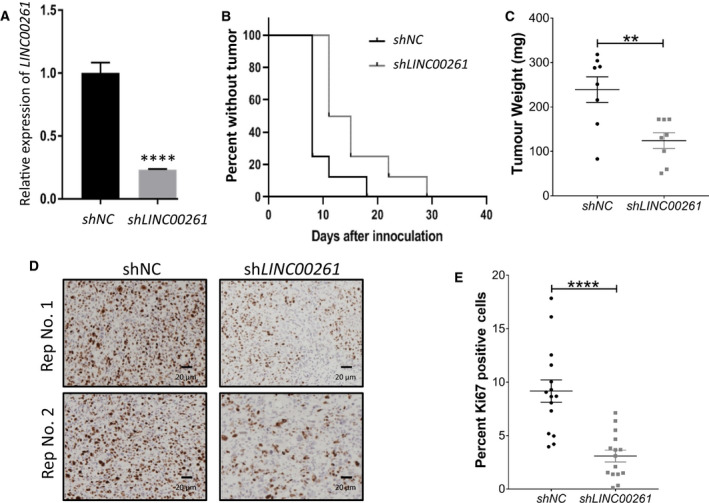
*LINC00261* silencing *in vivo* delays tumor formation. (A) *LINC00261* expression (qPCR) in PC‐3 cells stably expressing a nontargeting control (shControl) or shRNA targeting *LINC00261* (*P* < 0.0001). (B) Kaplan–Meier analysis for tumor formation in mice injected with cancer cells expressing sh*LINC00261* or sh*Control*. (C) Tumor weight of control or *LINC00261*‐silenced PC‐3 tumors upon harvest at day 32 after implantation into mice (**P = 0.0043). (D) Representative IHC images and (E) quantification of PC‐3 tumors from the control or *LINC00261*‐silenced arms stained for the proliferation marker Ki67. The scale bar at the bottom marks a 20 μm width. (A, C) Analyzed by unpaired two‐tailed *t*‐test. (B) Analyzed by log‐rank test: *P* = 0.018. (E) Images analyzed using imagej (see Materials and methods); statistically analyzed by unpaired two‐tailed *t*‐test *n* = 15. All data are presented as mean ± SEM with at least three independent biological replicates. ****P < 0.001.

Our experiments so far indicate that *LINC00261* promotes the proliferation and invasion of NEPC cells. To corroborate these findings *in vivo*, we generated PC‐3 cells with stable knockdown of *LINC00261* using a lentiviral shRNA system. After drug selection, we confirmed *LINC00261* knockdown in sh*LINC00261*‐treated cells (Fig. 3A) and, after a few passages, injected an equal number of viable sh*LINC00261* and shControl PC‐3 cells into both dorsal flanks of immunocompromised mice. Tumor growth was monitored twice per week for up to 32 days as previously described [28]. Notably, the median time to the formation of palpable tumors in individual mice was significantly delayed in *shLINC00261* vs shControl‐treated PC‐3 cells (log‐rank test: *P* = 0.018; Fig. 3B). After 32 days of growth, the average tumor weight was significantly smaller in *shLINC00261* relative to the control group (*P* = 0.0043; Fig 3C). Most notably, consistent with our *in vitro* data, the reduction in tumor weights was accompanied by a marked reduction in the levels of the proliferation marker Ki67 in the *shLINC00261* tumors (Fig. 3D,E). Taken together, these data strongly corroborate the functional requirement of *LINC00261* to sustain NEPC cell proliferation in a physiologically relevant *in vivo* system.

### 3.4 Nuclear *LINC00261* promotes cell invasion by regulating FOXA2 expression

**Fig. 4:**
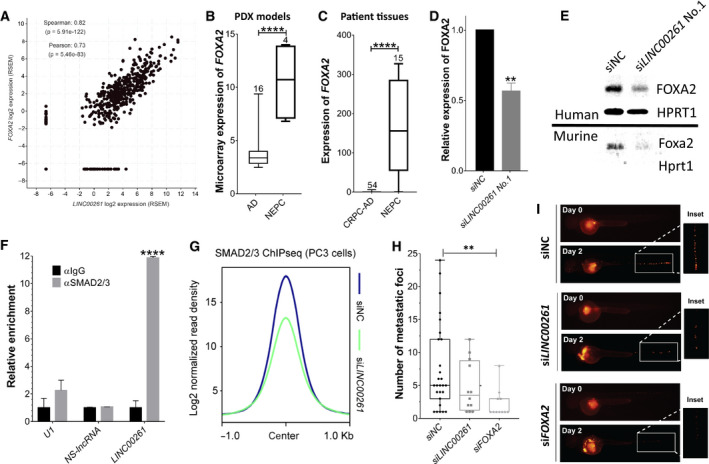
Nuclear *LINC00261* promotes transcription of FOXA2 to drive invasion in NEPC cells. (A) Correlation between *FOXA2* and *LINC00261* expression in transcriptomic data (RNA‐seq) from prostate adenocarcinoma patients (TANRIC database; Spearman's correlation = 0.82). (B) Relative expression (microarray) of *FOXA2* in several PDX models of prostate adenocarcinoma (*n* = 16) and NEPC (*n* = 4) from the Living Tumor Laboratory (**P = 0.0050). (C) Relative expression (qPCR) of *FOXA2* in FFPE biopsy samples from *t* NEPC (*n* = 34) and CRPC‐AD (*n* = 15) patients (Beltran *et al*. [16]; ****P < 0.0001). (D) *FOXA2* expression (qPCR) in PC‐3 cells transfected with a nontargeting control (siControl) or siRNA targeting *LINC00261* (**P = 0.0016). (E, top panel) Immunoblots showing FOXA2 protein expression in PC‐3 cells transfected with a nontargeting control (siControl) or siRNA targeting *LINC00261* (HPRT1 serves as loading control). (E, bottom panel) Expression of murine Foxa2 protein in NEPC OPT7714 cells transfected with a nontargeting control (siControl) or siRNA targeting *9030622O22‐Rik*. (F) Relative quantification (qPCR) of *U1, NS‐lncRNA*, and *LINC00216* transcripts in RIP experiments using anti‐IgG or anti‐SMAD2/3 antibody in PC‐3 cells. (G) SMAD2/3 ChIP‐seq peak profile plot averaged from 6724 binding sites in PC‐3 cells treated with either the nontargeting control siRNA (siNC) or siRNA targeting the *LINC00261* (si*LINC00261*) gene. (H) The number of distinct metastatic foci in the body of zebrafish embryos injected with PC‐3‐RFP cells with or without *LINC00261* or FOXA2 knockdown (*P* = 0.0146). (I) Representative images of zebrafish embryos from panel H. (B, C) Data Analyzed by two‐tailed unpaired *t*‐test. (B) Data obtained from the living tumor laboratory. (C) RNA‐seq data were obtained from the public database cBioPortal using the Trento NEPC dataset. (D) Analyzed by two‐tailed *t*‐test. (F) Analyzed by one‐way ANOVA with the Siddak *post hoc* test. (H) Analyzed by two‐way ANOVA with Dunnett's *post hoc* test. All data are shown as mean ± SEM and are from at least three biological replicates.

As most lncRNAs work with cognate protein partners to have a functional effect, we interrogated the TANRIC database [29] to identify protein‐coding genes associated with *LINC00261* expression in patient tumors. We found FOXA2 to be the most significantly co‐expressed gene with *LINC00261* (Spearman's correlation = 0.82; Fig. 4A). This positive transcriptional correlation was evident even in other cancers (Table S2), and in normal tissues, both LINC00261 and FOXA2 were highly co‐expressed in endoderm‐derived organs (Fig. S4A,B). Concordant with the clinical expression profile of *LINC00261*, *FOXA2* expression was significantly elevated in NEPC PDX models (Fig. 4B) and NEPC patient tumors (Fig. 4C) compared with prostate adenocarcinoma. *FOXA2* expression was also significantly elevated in metastatic PCa lesions from the liver (Fig. S4C). Intriguingly, after castration in mice bearing the LTL‐331 tumor line, FOXA2 expression was followed by the upregulation of *LINC00261*, peaking at about 12wks, as the tumor cells transdifferentiate into the neuroendocrine LTL‐331R tumor line (Fig. S4D). This is accompanied by specific activation of FOXA2 target genes in LTL‐331R (Fig. S4E). *LINC00261* is coded just 2.4 kb downstream of FOXA2 on chromosome 20, and a recent study showed *LINC00261* to physically recruit SMAD2/3 to the FOXA2 promoter coded *in cis* [30]. Thus, we set to validate whether a similar mechanism couples together the expression of *LINC00261* and FOXA2 in NEPC cells.

Firstly, we knocked down *LINC00261* in PC‐3 cells and found a parallel downregulation in both the mRNA (Fig. 4D) and protein abundance of FOXA2 (Fig. 4E). Interestingly, we found *9030622O22‐RiK* to similarly regulate Foxa2 expression in the murine NEPC cell line OPT7714 (Fig. 4E). Next, we performed RNA co‐immunoprecipitation (RIP) assays using an anti‐SMAD2/3 or IgG control antibody in PC‐3 cells. Using qPCR, we detected a specific and dramatic (> 12‐fold) co‐enrichment of *LINC00261* upon SMAD2/3 immunoprecipitation relative to the IgG control (Fig. 4F, *P* < 0.0001). In ChIP followed by sequencing (ChIP‐seq) assay, *LINC00261* knockdown triggered a marked decrease in the chromatin binding of the SMAD2/3 transcriptional complex (Fig. 4G, Fig. S5A), including SMAD2/3 binding at *cis*‐regulatory elements of the *FOXA2* gene and bona fide TGF‐β1/SMAD target genes, namely *ROR1*, *SKIL*, and *SPTLC3* (Fig. S5A,B). Thus, *LINC00261* regulates FOXA2 mRNA expression by recruiting the SMAD transcriptional machinery to the *cis*‐regulatory elements of the *FOXA2* gene in NEPC cells, which is consistent with previous reports describing SMAD‐mediated regulation of FOXA2 expression in other cell lineages [30, 31, 32].

This prompted a related question: Which oncogenic functions of *LINC00261* are mediated through FOXA2? To address this, using siRNAs we knocked down *FOXA2* expression in PC‐3 cells and assessed the impact on proliferation and invasion. While knockdown of *FOXA2* had no effect on cellular proliferation, it dramatically attenuated the ability of PC‐3 cells for anchorage‐independent growth in the soft agar colony formation assay (Fig. S5C). It also dramatically attenuated the metastatic spread of PC‐3 cells in zebrafish embryos. In this assay, we treated RFP‐labeled PC‐3 cells with a control or *FOXA2* or *LINC00261* targeting siRNA and injected equal number of viable cells into the perivitelline space of zebrafish embryos at 2 days postfertilization (*n* > 40 in each group) as previously described [33]. Injected embryos were monitored for invasion and spread of PC‐3 cells into the body using fluorescent microscopy up to 7 days of age. Notably, knockdown of *FOXA2* dramatically attenuated the rate (Fig. S5D,E) and the extent of metastatic dissemination (Fig. 4H,J) of PC‐3 cells relative to the control group, which was comparably phenocopied by the knockdown of *LINC00261* alone. Altogether, these findings suggest that nuclear upregulation of *LINC00261* activates FOXA2 expression *via* chromatin recruitment of the SMAD2/3 complex in NEPC cells, and FOXA2 in turn drives a gene program of anchorage‐independent survival and growth at the metastatic site.

### 3.5 Cytoplasmic *LINC00261* promotes proliferation by regulating CXB2 expression

**Fig. 5:**
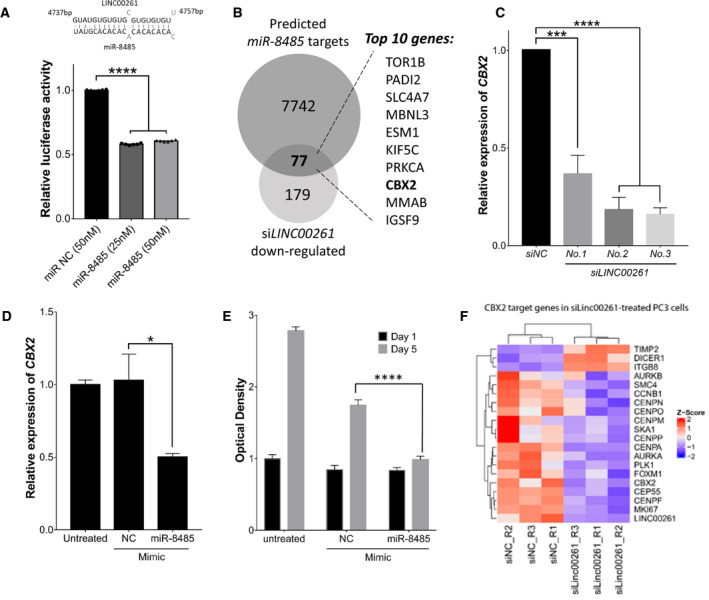
Cytoplasmic *LINC00261* buffers miR‐8485 increasing CBX2 activity to drive proliferation in NEPC cells. (A, top) Computational analyses predict *miR‐8485* binding within 3′UTR of *LINC00261*. The partially complementary seed sequence is visualized below. (A, bottom) Luciferase activity from the *LINC00261*‐binding reporter assay with transfection of *miR‐8485* or a nontargeting control miRNA mimic in HEK293 cells. For each treatment group, reporter activity was normalized to background signals from the unmodified pmirGlo reporter alone. (B) Overlap between predictive targets of *miR‐8485* (*n* = 7742) and differentially expressed genes (DEGs; *n* = 179) in *LINC00261*‐silenced PC‐3 cells (RNA‐seq). 77 gene candidates were common in the two lists with top 10 genes shown. (C) CBX2 expression (qPCR) in PC‐3 cells treated with a nontargeting control (siControl) or siRNA targeting *LINC00261* (***P = 0.0002; ****P < 0.0001) or (D) treated with *miR‐8485* or nontargeting miRNA mimic (*P = 0.0434). (E) Viability (MTT assay) of PC‐3 cells treated with a negative control mimic (NC mimic) or *miR‐8485* mimic (****P < 0.0001). (F) Heat map showing mRNA expression (RNA‐seq) changes in CBX2 target genes in PC‐3 cells treated with si*LINC00261* or siNC. (A, B) Analyzed by two‐tailed *t*‐test *n* = 6. (D) Analyzed by one‐way ANOVA with Dunnett's *post hoc* test *n* = 2. (E) Analyzed by two‐tailed *t*‐test *n* = 3. (F) Analyzed by two‐way ANOVA with Tukey's *post hoc* test *n* = 6. All data are shown as mean ± SEM.

A predominant fraction of *LINC00261* is retained in the cytoplasm of PC‐3 cells (Fig. 2B), where several lncRNAs have been shown to function as a miRNA sponge [34]. Thus, using publically available computational tools and miRNA databases, we explored whether *LINC00261* had binding sites for known miRNAs. Here, in many independent analyses, we found a high‐confidence binding site for the miRNA called *miR‐8485* within the 3′ UTR of *LINC00261* (top, Fig. 5A). Thus, we set out to experimentally validate the binding of *miR‐8485* to *LINC00261* using the luciferase reporter assay.

We aligned *miR‐8485* to the *LINC00261* and mapped the 100‐bp segment in the 3′ UTR centered at the miRNA‐binding site. We cloned this segment into the 3′ UTR of the firefly luciferase gene or cloned the same segment without the 20bp *miR‐8485* seed region to use as a negative control reporter. These reporter vectors were co‐transfected with a *miR‐8485* mimic or a negative control miRNA in HEK293 cells, and the luciferase reporter activity was measured. Here, we found *miR‐8485* overexpression to result in ~ 2‐fold reduction in reporter activity relative to cells treated with the control miRNA (bottom, Fig. 5A). As expected, this regulation was completely abolished for the control reporter where the 20bp *miR‐8485* seed region was deleted (Fig. S6A). This raised a pertinent question: Which gene targets of *miR‐8485* are buffered by the *LINC00261* transcript? To address this, using RNA‐seq we profiled transcriptomic changes upon *LINC00261* knockdown in PC‐3 cells (Table S3) and overlapped the differentially expressed gene list with computationally predicted targets of *miR‐8485* (see Materials and methods). This identified 77 distinct genes, which notably included a known epigenetic driver of NEPC called CBX2 (Fig. 5B, Table S4; [35, 36]). Notably, knockdown of *LINC00261* led to a dramatic reduction in CBX2 expression (Fig 5C). Consistently, the overexpression of *miR‐8485* reduced the expression of CBX2, while exogenous overexpression of an antisense *miR‐8485* inhibitor increased the expression of the CBX2 transcript (Fig. 5D, Fig. S6B). Long‐term treatment with the *miR‐8485* mimic also led to a significant inhibition of cell proliferation in PC‐3 cells (Fig. 5E). Next, to assess transcriptional activity of CBX2 in *LINC00261*‐silenced PC‐3 cells, we profiled changes in the expression of CBX2 target genes previously identified in this study [36]. We found a dramatic concordance between si*LINC00261* and si*CBX2* gene signatures, with several CBX2 upregulated genes being downregulated upon *LINC00261* knockdown and *vice versa* (Fig. 5F). Furthermore, we corroborated that *miR‐8485* levels were reduced in NEPC using the LTL313 xenograft model upon castration (Fig. S6C) and confirmed CBX2 expression was increased in metastatic advanced PCa tissue with NEPC features (Fig. S6D). Altogether, these findings show that the cytoplasmic fraction of *LINC00261* binds to and sequesters *miR‐8485*, which otherwise inhibits CBX2 activity and thereby proliferation in NEPC cells.

## 4 Discussion

Neuroendocrine prostate cancer is a universally fatal variant of PCa due to its late diagnosis, treatment resistance, and metastatic capability. Hence, new therapeutic approaches are urgently needed for this disease. Given increasing evidence implicating lncRNAs in cancer biology, we hypothesized that the highly conserved lncRNAs may play a role in the initiation and progression of NEPC. Identification of lncRNAs relevant for NEPC may provide an untapped resource of potential therapeutic targets in the fight against this rapidly fatal disease. Previous studies have used bioinformatics [37] or candidate gene approaches [38] to explore the noncoding transcriptome of NEPC. In this study, we conducted an unbiased, whole‐transcriptome analysis, which allowed us to identify and functionally characterize *LINC00261*, a highly conserved lncRNA that drives NEPC proliferation and survival. Evolutionarily conserved lncRNAs are often implicated in fundamental cellular functions and control the neoplastic transformation of different tissues and organisms [13]. This paradigm is corroborated by the second most conserved lncRNA in our list, *MIAT*. *MIAT* is known to be expressed in NEPCs and is correlated with poor prognosis, high metastatic potential, and frequent *Rb* mutations [39]. Notably, *MIAT's* oncogenic role has been confirmed in other malignancies [40], and this lncRNA plays disparate roles in neural tissue specification [41] and vascular remodeling [42].

Our results, as well as data from independent databases, confirmed that *LINC00261* is highly upregulated in clinical NEPC samples. Our analyses also revealed that *LINC00261* expression is negatively correlated with AR amplification in clinical samples and is restricted to AR‐negative preclinical models; this suggested that this *LINC00261* is crucial for the survival of androgen‐indifferent cells. This hypothesis was corroborated by our *in vitro* and *in vivo* experiments on cell proliferation and tumor cell metastasis. Recent studies indicate that androgen‐indifferent PCas often activate several alternative survival pathways [43]. This paradigm is in agreement with the positive correlation between *AKT2* amplification and *LINC00261* expression in PCa clinical samples.

Notably, *LINC00261* is also upregulated in hepatic metastases from prostate adenocarcinomas. This observation could be explained by the fact that the hepatic micro‐environment promotes the neuroendocrine differentiation of PCa cells [23, 44]. Since liver metastases are associated with a particularly dismal prognosis [23], our findings further emphasize the clinical relevance of *LINC00261*.

It has been previously shown that *LINC00261* recruits SMAD2/3 to the promoter of *FOXA2*, thereby inducing its transcription in normal cells [30]. Here, we have shown that *LINC00261* directly binds to and recruits the SMAD2/3 complex to *cis*‐regulatory elements of *FOXA2*, thereby upregulating FOXA2 expression in neuroendocrine‐like PCa cells. Notably, in the genomic space *FOXA2* and *LINC00261* are coded in immediate genomic proximity of each other and are co‐expressed across cell lineages, raising an intriguing possibility of functionally related lncRNA and protein‐coding gene pairs to show a higher degree of synteny through evolution. FOXA2 is a pioneer transcription factor, which is able to bind compacted chromatin and make it accessible for transcription [45]. FOXA2 had been reported as a selective marker for NEPC [46], but its role in metastasis and molecular interactions in this context have not been fully elucidated. Interestingly, we have previously shown that global alterations of the chromatin structure underpin the transdifferentiation from prostatic adenocarcinoma to NEPC [7]. Based on our findings and on previous evidence, it is conceivable that FOXA2 plays a pivotal role in the chromatin remodeling of NEPC cells. FOXA2 has also been shown to promote the recruitment of p300 to transactivate hypoxia‐inducible factor (HIF)—regulated genes, which are highly expressed by metastatic PCa. These genes are required for the formation of NEPCs in siah2‐null TRAMP mouse models [47]. Our *in vivo* data indicate that *LINC00261* upregulation precedes *FOXA2* expression during the transdifferentiation process and that this event is followed by the activation of the FOXA2 transcriptional program. Notably, at least two of the FOXA2 transcriptional targets identified by our analysis (*ANK2* and *RAB3C*; Fig. S4C) are known to promote cancer progression and metastasis [48, 49].

CBX2 is one of the most highly upregulated epigenetic modifiers in the neuroendocrine disease and in cancer cells is required for cellular proliferation [35, 36, 50]. Our findings reveal a novel *miR‐8485*/CBX2 regulatory axis in PC‐3 cells and show that *LINC00261* directly binds to and sequesters *miR‐8485* (i.e., works as a miRNA sponge), thereby increasing CBX2 activity that fuels the growth of NEPC. Past studies have shown CBX2 activity in cancer to be regulated by miRNAs [51], and to this list, we add a novel candidate *miR‐8485* from PCa cells. There could be other oncogenic targets of *miR‐8485* in NEPC, and future studies are needed to uncover these genes and their role in disease biology.

## 5 Conclusions

**Fig. 6:**
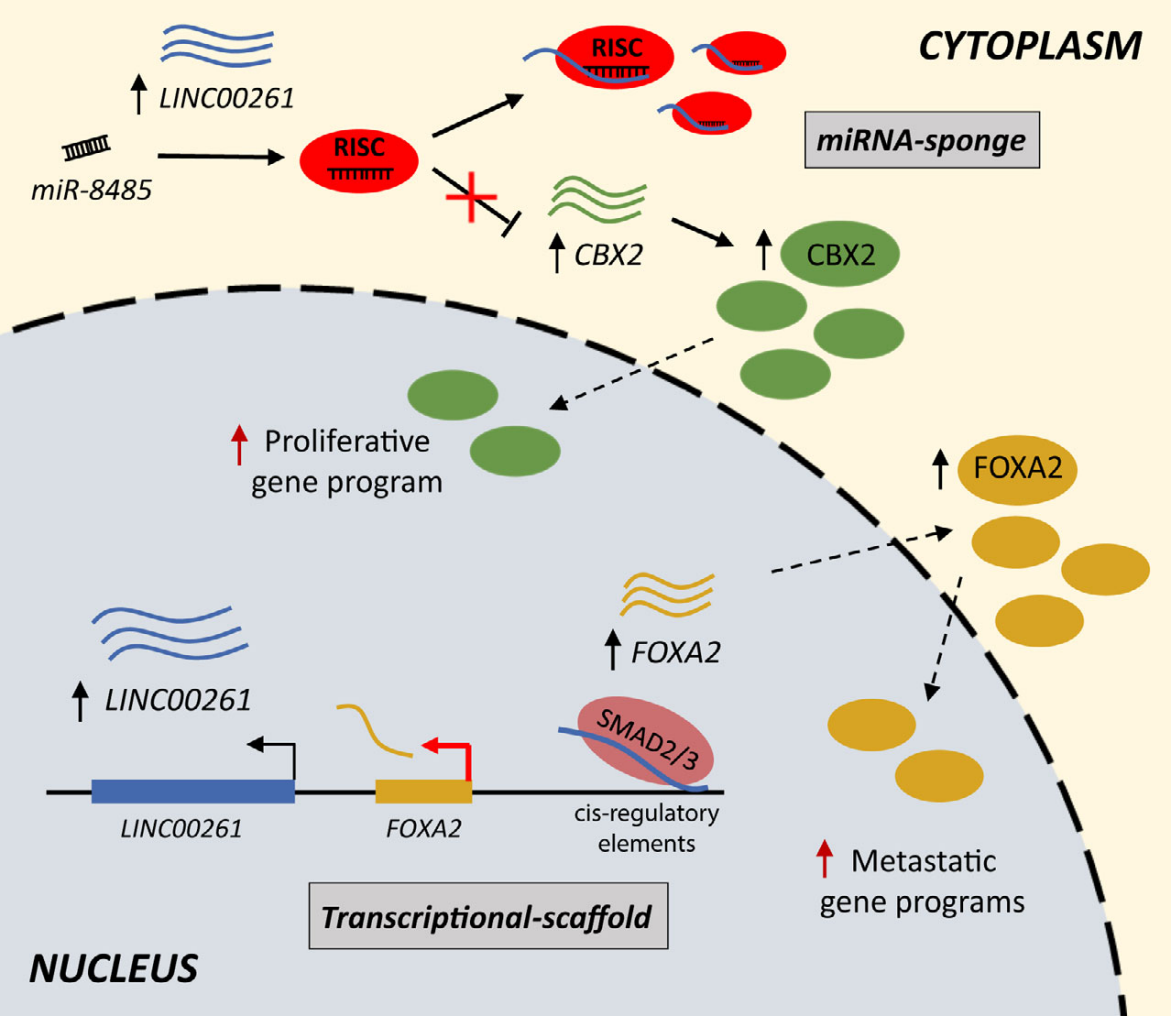
Cell compartment‐specific oncogenic functions of *LINC00261*. Inside the nucleus, *LINC00261* functions as a transcriptional scaffold and recruits the SMAD2/3 transcriptional machinery to the FOXA2 cis‐regulatory elements, thereby increasing the FOXA2‐driven anchorage‐independent growth and metastatic gene programs. In the cytoplasm, *LINC00261* sequesters the *miR‐8485:*RISC complex to block its binding with the *CBX2* transcript, thereby increasing the CBX2‐driven proliferative gene program. Thus, *LINC00261* orchestrates neuroendocrine trans‐differentiation in PCa by regulating CBX2 and FOXA2 activities *via* distinct, cellular compartment‐specific molecular mechanisms. RISC, RNA‐induced silencing complex.

In summary, we identify a highly conserved *LINC00261* to be centrally involved in NEPC pathogenesis by directly upregulating the expression of CBX2 and FOXA2, which independently function as key epigenetic drivers of hyperproliferation and metastasis, respectively. Most importantly, we delineate two distinct, cellular compartment‐specific roles of *LINC00261*. In the nucleus, *LINC00261* acts as a transcriptional scaffold to recruit the SMAD transcriptional machinery at the *FOXA2 cis*‐regulatory elements, while in the cytoplasm, *LINC00261* binds to and sequesters miR‐8485 that targets *CBX2* (Fig. 6). While no lncRNA‐targeting drug has been approved so far, recent reports indicate that antisense oligonucleotides (ASOs) targeting oncogenic lncRNAs are effective anticancer agents *in vivo* [52]. Notably, ASOs are in clinical trials for the treatment of several pathologies [53]. Hence, *LINC00261* may represent an attractive therapeutic target against metastatic NEPC and could provide a novel route to inhibit disease‐driving chromatin modifiers that are currently considered undruggable.

## Conflict of interest

The authors declare no conflict of interest.

## Author contributions

RLM, AP, and FC conceived and designed the study. RLM and AP conducted most of the experiments with the assistance of SEC, EV, DRC, MJ, S‐CC, PP, and XC. RW, HX, and DL conducted the *in vivo* experiments. NN, IA, and IU conducted *in silico* computational analyses of multi‐omics data and evolutionary conservation of long intergenic noncoding RNA (lincRNAs). EJ and MPC carried out experiments involving murine cells. YL and WJ performed immunoprecipitation experiments. CH helped with immunoblotting experiments. CM was involved in lncRNA overexpression studies. LQ and HP were involved in analyses and interpretation of clinical and transcriptomic data. IR helped perform invasion and migration experiments. RLM, AP, and FC supervised the study and wrote the manuscript. RML, AP, and SEC made and arranged all the figures. All authors read and critically revised the paper.

## Supporting information


**Fig. S1**. LncRNAs up‐regulated in the PDX LTL‐331R, ranked by evolutionary conservation in 17 species. (A) Workflow for lncRNA identification. Transcripts were identified by RNA‐seq of the patient‐derived xenograft (PDX) models LTL‐331 (prostatic adenocarcinoma) and LTL‐331R (NEPC). lncRNA reads were shortlisted with the criteria log_2_ fold change < −4; false‐discovery rate < 0.1; fragments per kilobase of transcript per million mapped reads‐FPKM > 10. (B) Ranked shortlist of lncRNAs ordered by number of species with orthologs in Hezroni *et al*., 2015. NEPC Associated lncRNAs (*NEARs*) were then assigned numbers based on their rank in this shortlist.

**Fig. S2**. Subcellular localization of murine ortholog *9030622O22‐Rik* and essentiality of *LINC00261* in NEPC. (A) The expression of *LINC00261*'s murine ortholog *9030622O22‐Rik* in T23 (murine prostatic adenocarcinoma) and OPT7714 (murine NEPC). (B) Subcellular localization of *Gapdh, Malat1*, and *9030622O22‐Rik* RNA transcripts in OPT7714 cells. (C) Expression of *LINC00261* after treatment with *LINC00261*‐targeting siRNAs in PC‐3 cells (siRNA No. 1 *P* < 0.0001 siRNA No. 2 *P* < 0.0001, siRNA No. 3 *P* < 0.0001, all relative to siNC treated cells). (D) Representative images of PC‐3 cells 72 h after transfection with *siNC* or si*LINC00261* (E) Viable PC‐3 cell counts after treatment with a control siRNA or three different si*LINC00261* for 3 days. (F) Viable DU‐145 cells 7 days after treatment with negative control empty vector (NC) or *LINC00261* overexpression vector (overexpression‐OE; **P = 0.0030). (A, B) data *n* = 3, ± SEM, Analyzed by graphpad prism 7 software. (C) Analyzed by one‐way ANOVA with Dunnett's *post hoc* test. (E) Analyzed by unpaired two‐tailed *t*‐test (*n* = 2). All data Analyzed using graphpad prism 7 software, *n* = 3 ± SEM unless otherwise stated.

**Fig. S3**. Effects of *LINC00261* on metastatic cellular properties. (A) *LINC00261* expression (qPCR) in PC‐3 cells at 18 h post transfection with a nontargeting control (siControl) or siRNAs targeting *LINC00261*. (B) Viability (MTT assay) in PC‐3 cells 18 h post treatment with a negative control mimic (NC mimic) or distinct siRNA targeting *LINC00261*. (C) Rate of wound healing in PC‐3 cells with or without *LINC00261* knock‐down. (D) Representative wound‐healing images taken at 0, 8, and 24 h (PC‐3 cells). (A, B) Analyzed by one‐way ANOVA with Dunnett's multiple comparisons test. All statistical analysis Analyzed using graphpad prism 7 software, *n* = 3 ± SEM.

**Fig. S4**. *FOXA2* expression and activity is associated with NEPC. (A) Expression (qPCR) of *LINC00261* in the NEPC PDX mode LTL‐331Rl and a panel of non‐neoplastic tissues (****P < 0.0001). Numbers above bars represent the number of samples pooled. (B) Expression (qPCR) of *FOXA2* in the NEPC PDX model LTL‐331R and a panel of non‐neoplastic tissues (****P < 0.0001). Numbers above bars represent the number of samples pooled. (C) Expression of FOXA2 in clinical samples of secondary PCa lesions from primary site/lymph nodes (*n* = 53), bone (*n* = 29) or liver (*n* = 17) (****P < 0.0001, ANOVA and Tukey *post hoc* test). Data from cBioPortal, Metastatic PCa SU2C/PCF Dream Team Cell 2015 (D) Time‐lapse expression of *LINC00261* and FOXA2 (RNA‐seq) in the LTL‐331/331R PDX models post‐Cx: postcastration. (E) Heat map showing expression changes in the AR and FOXA2 transcriptional programs in the LTL331/331R NE trans‐differentiation PDX models. Top panel ‐ AR signaling targets are up‐regulated in LTL‐331; middle panel‐ NE markers are up‐regulated in LTL‐331R; bottom panel FOXA2 transcriptional targets (validated using www.amp.pharm.mssm.edu) are up‐regulated in LTL‐331R. Data obtained from RNA‐seq data of three individual samples for each LTL model. Blue tones lower expression; red tones higher expression. (A–C) Analyzed by one‐way ANOVA with Dunnett's *post hoc* test (*P = 0.0277). RNA‐seq data were obtained from the public database cBioPortal using the Trento NEPC dataset. (E) Data visualized by Microsoft excel. All data ± SEM except E (min to max). All statistical analysis Analyzed using graphpad prism 7 software.

**Fig. S5**. *LINC00261* knockdown attenuates SMAD2/3 chromatin binding and hinders anchorage‐independent growth and metastatic ability of PC‐3 cells. (A) Top, Normalized SMAD2/3 ChIP‐seq read densities at all of its genomic binding sites (*n* = 6724) in PC‐3 cells or, bottom, cis‐regulatory genomic sites within the 4Mb window centered at the FOXA2 gene (*n* = 10). (B) SMAD2/3 ChIP‐seq read density tracks from siNC or si*LINC00261*‐treated PC‐3 cells at distinct genomic loci encoding bona fide TGF‐β1/SMAD target genes. (C) Left, Representative images of iodonitrotetrazolium chloride stained soft agar PC‐3 cell colonies after treatment with either the nontargeting, control siRNA (siNC) or siRNA targeting the *LINC00261* (si*LINC00261*) transcript (two‐tailed *t*‐test). Insets at the bottom show magnified images of the colonies. Right, Average number of soft agar colonies per 10× field of siNC or si*LINC00261*‐treated PC‐3 cells. Distinct cells colonies were counted from three randomly chosen 10X field per well from two biological replicates. (D) Expression (qPCR) of *LINC00261* and FOXA2 in siRNA‐treated PC‐3‐RFP cells used for the in zebrafish metastasis experiments. (E) Percentage of fish that show metastatic dissemination at 1 and 2 days after injection with PC‐3‐RFP cell treated with a nontargeting control (siControl; *n* = 52), siRNA targeting *LINC00261* (*n* = 40), or siRNA targeting *FOXA2* (*n* = 44). (A, D) was statistically analyzed using two‐tailed *t*‐test and (C) was analyzed using a one‐way ANOVA with Dunnett's *post hoc* test.

**Fig. S6**. *miR‐8485* mimic downregulates the expression of CBX2 to enable NEPC progression. (A) Luciferase activity from the control, mutated *LINC00261* binding reporter assay (see Materials and methods) with transfection of *miR‐8485* or a nontargeting control miRNA mimic in HEK293 cells. For each treatment group, reporter activity was normalized to background signals from the unmodified pmirGlo reporter alone. (B) Expression of CBX2 is significantly reduced upon expression of miR‐8485 mimic compared to mimic control. Expression of CBX2 is significantly increased upon miR‐8485 inhibition compared to inhibitor control. (C) Expression of has‐miR‐8485 in CRPC‐Adeno (*n* = 13) or NEPC (*n* = 4) patient‐derived PCa xenografts that are hormone‐sensitive or hormone‐resistant. (D) Expression of CBX2 in PCa patient samples with (*n* = 15) or without (*n* = 34) presentation of NEPC features. (B) Data analyzed by unpaired two‐tailed *t*‐test (*P < 0.05).

**Table S1**. *LINC00261* is associated with NEPC markers and *FOXA2*. Using co‐expression analysis on cBioPortal accessed from the Trento dataset *LINC00261* expression has a tendency towards co‐occurrence with *FOXA2,* and the NEPC markers *ENO2, CHGA, CBX5* and *NCAM1* expression. There is also a tendency towards co‐occurrence between *FOXA2* and the markers *ENO2, CHGA, CBX5* and *NCAM1*.

**Table S2**. *LINC00261* survival data from all other cancers available on the TANRIC database with the top correlated mRNA in each dataset. Using the TANRIC database each cancer type was queried for *LINC00261* expression correlated with survival, and with mRNA. Only the top Spearman score mRNA is shown per dataset. Numbers of clinical samples in each data set is shown.

**Table S3**. List of differentially expressed genes (RNA‐seq) in si*LINC00261*‐treated PC‐3 cells. RNA‐seq analysis was carried out using criteria described in Materials and methods section. In total, the analyses revealed 180 down‐regulated transcripts, and 519 up‐regulated transcripts upon *LINC00261* knock‐down.

**Table S4**. Common genes between the predicted miR‐8485 targets and genes down‐regulated upon *LINC00261* silencing in PC‐3 cells. For each of the 77 genes, fold change and associated *P*‐value upon *LINC00261* silencing are shown, along with identification of the miRNA‐binding tools that predict targeting by miR‐8485.

## Data Availability

All data generated or analyzed during this study are included in this published article and its supplementary information files.
