# Supplementary figures and images for: The evolutionarily conserved long non‐coding RNA LINC00261 drives neuroendocrine prostate cancer proliferation and metastasis via distinct nuclear and cytoplasmic mechanisms
Source: Mol Oncol. 2021 Apr 26;15(7):1921–41. doi: 10.1002/1878-0261.12954 (PMC8253100; doi:10.1002/1878-0261.12954)

## Slide 1
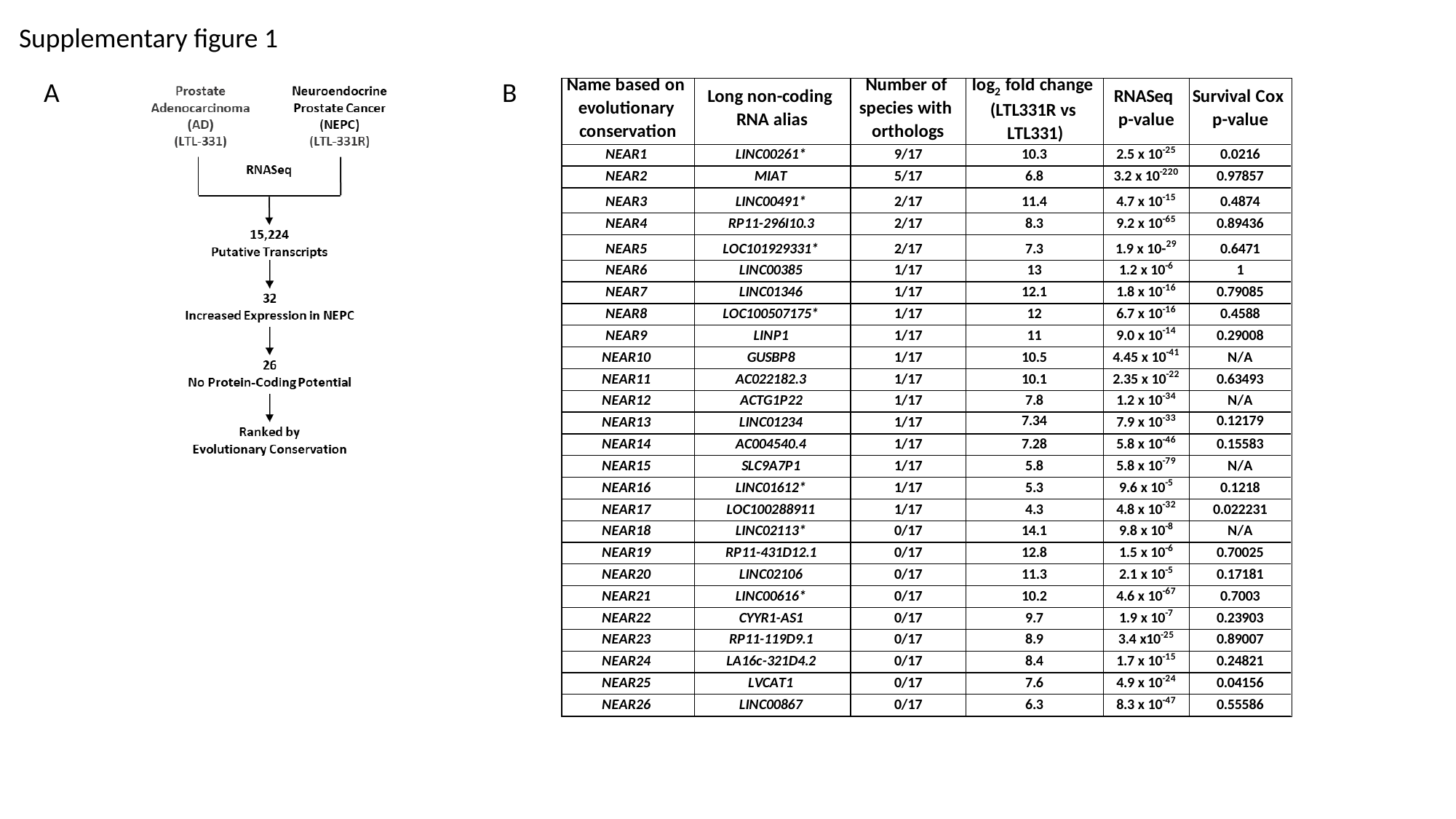

Supplementary figure 1
A
B

Supplement: Supplementary file 1 — Fig. S1. LncRNAs up‐regulated in the PDX LTL‐331R, ranked by evolutionary conservation in 17 species. (A) Workflow for lncRNA identification. Transcripts were identified by RNA‐seq of the patient‐derived xenograft (PDX) models LTL‐331 (prostatic adenocarcinoma) and LTL‐331R (NEPC). lncRNA reads were shortlisted with the criteria log2 fold change < −4; false‐discovery rate < 0.1; fragments per kilobase of transcript per million mapped reads‐FPKM > 10. (B) Ranked shortlist of lncRNAs ordered by number of species with orthologs in Hezroni et al., 2015. NEPC Associated lncRNAs (NEARs) were then assigned numbers based on their rank in this shortlist. [file MOL2-15-1921-s001.pptx]

## Slide 1
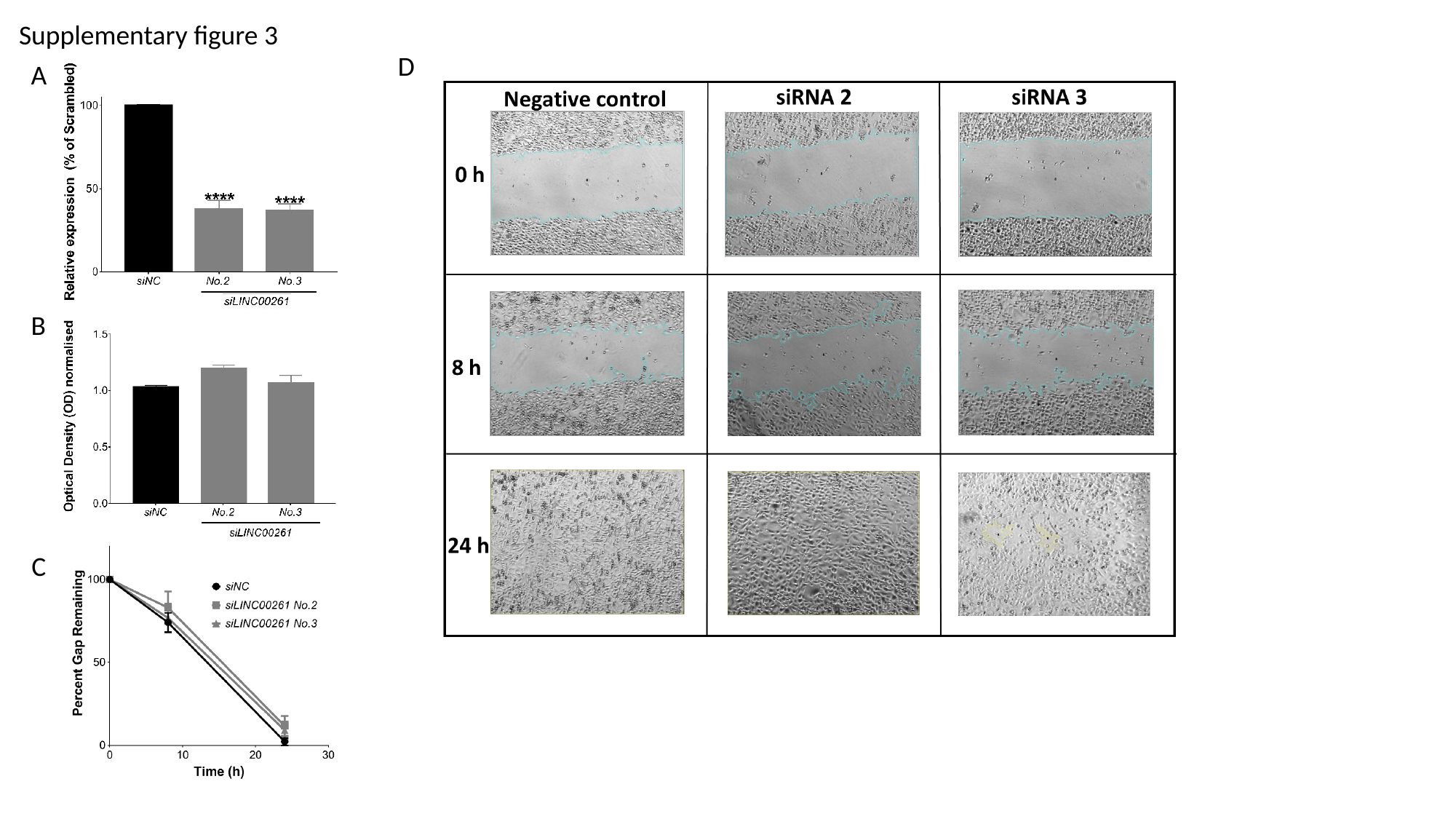

Supplementary figure 3
D
A
B
C

Supplement: Supplementary file 3 — Fig. S3. Effects of LINC00261 on metastatic cellular properties. (A) LINC00261 expression (qPCR) in PC‐3 cells at 18 h post transfection with a nontargeting control (siControl) or siRNAs targeting LINC00261. (B) Viability (MTT assay) in PC‐3 cells 18 h post treatment with a negative control mimic (NC mimic) or distinct siRNA targeting LINC00261. (C) Rate of wound healing in PC‐3 cells with or without LINC00261 knock‐down. (D) Representative wound‐healing images taken at 0, 8, and 24 h (PC‐3 cells). (A, B) Analyzed by one‐way ANOVA with Dunnett's multiple comparisons test. All statistical analysis Analyzed using graphpad prism 7 software, n = 3 ± SEM. [file MOL2-15-1921-s006.pptx]

## Slide 1
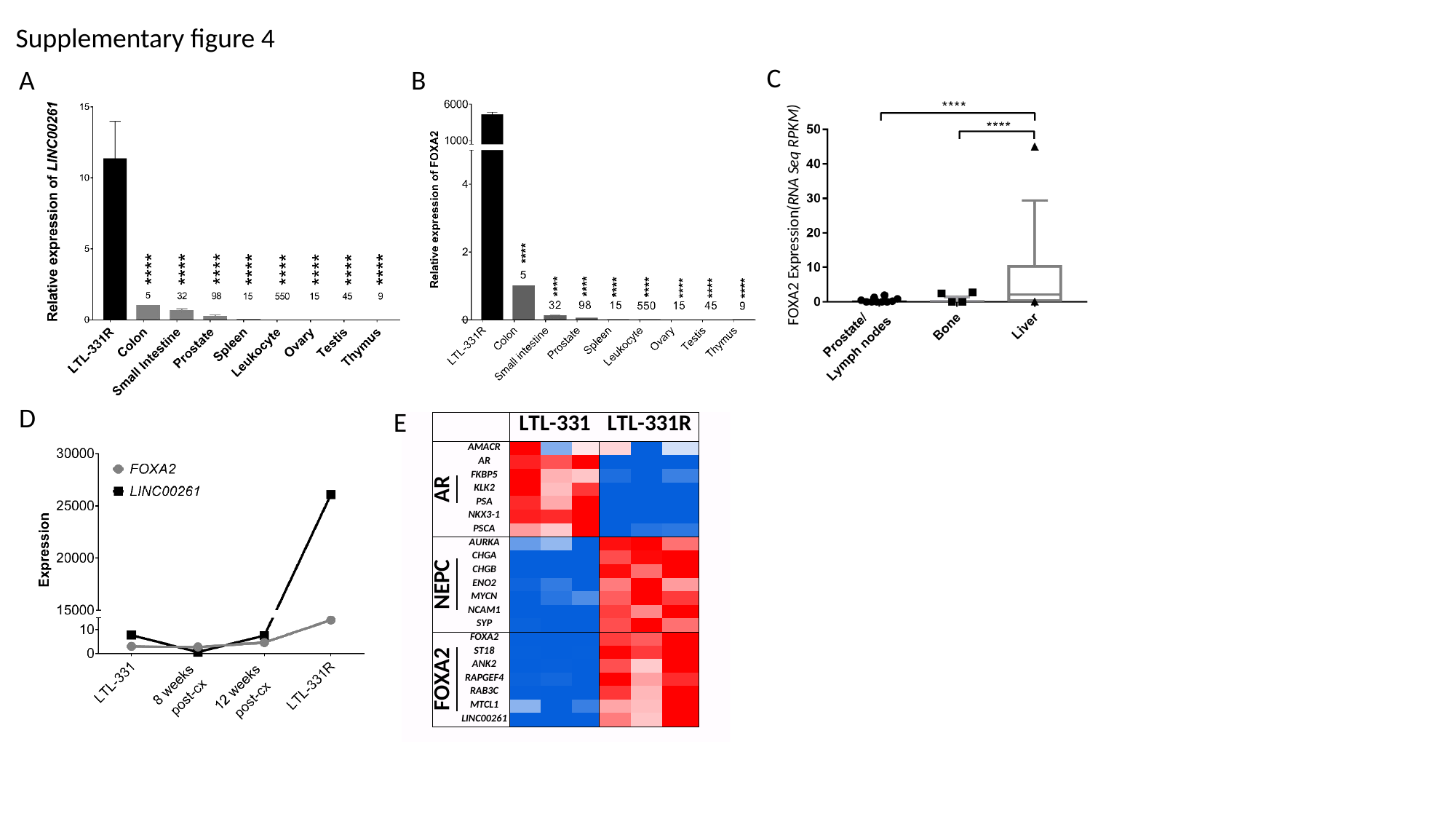

Supplementary figure 4
C
A
B
FOXA2 Expression(RNA Seq RPKM)
D
E

Supplement: Supplementary file 4 — Fig. S4. FOXA2 expression and activity is associated with NEPC. (A) Expression (qPCR) of LINC00261 in the NEPC PDX mode LTL‐331Rl and a panel of non‐neoplastic tissues (****P < 0.0001). Numbers above bars represent the number of samples pooled. (B) Expression (qPCR) of FOXA2 in the NEPC PDX model LTL‐331R and a panel of non‐neoplastic tissues (****P < 0.0001). Numbers above bars represent the number of samples pooled. (C) Expression of FOXA2 in clinical samples of secondary PCa lesions from primary site/lymph nodes (n = 53), bone (n = 29) or liver (n = 17) (****P < 0.0001, ANOVA and Tukey post hoc test). Data from cBioPortal, Metastatic PCa SU2C/PCF Dream Team Cell 2015 (D) Time‐lapse expression of LINC00261 and FOXA2 (RNA‐seq) in the LTL‐331/331R PDX models post‐Cx: postcastration. (E) Heat map showing expression changes in the AR and FOXA2 transcriptional programs in the LTL331/331R NE trans‐differentiation PDX models. Top panel ‐ AR signaling targets are up‐regulated in LTL‐331; middle panel‐ NE markers are up‐regulated in LTL‐331R; bottom panel FOXA2 transcriptional targets (validated using www.amp.pharm.mssm.edu) are up‐regulated in LTL‐331R. Data obtained from RNA‐seq data of three individual samples for each LTL model. Blue tones lower expression; red tones higher expression. (A–C) Analyzed by one‐way ANOVA with Dunnett's post hoc test (*P = 0.0277). RNA‐seq data were obtained from the public database cBioPortal using the Trento NEPC dataset. (E) Data visualized by Microsoft excel. All data ± SEM except E (min to max). All statistical analysis Analyzed using graphpad prism 7 software. [file MOL2-15-1921-s010.pptx]

## Slide 1
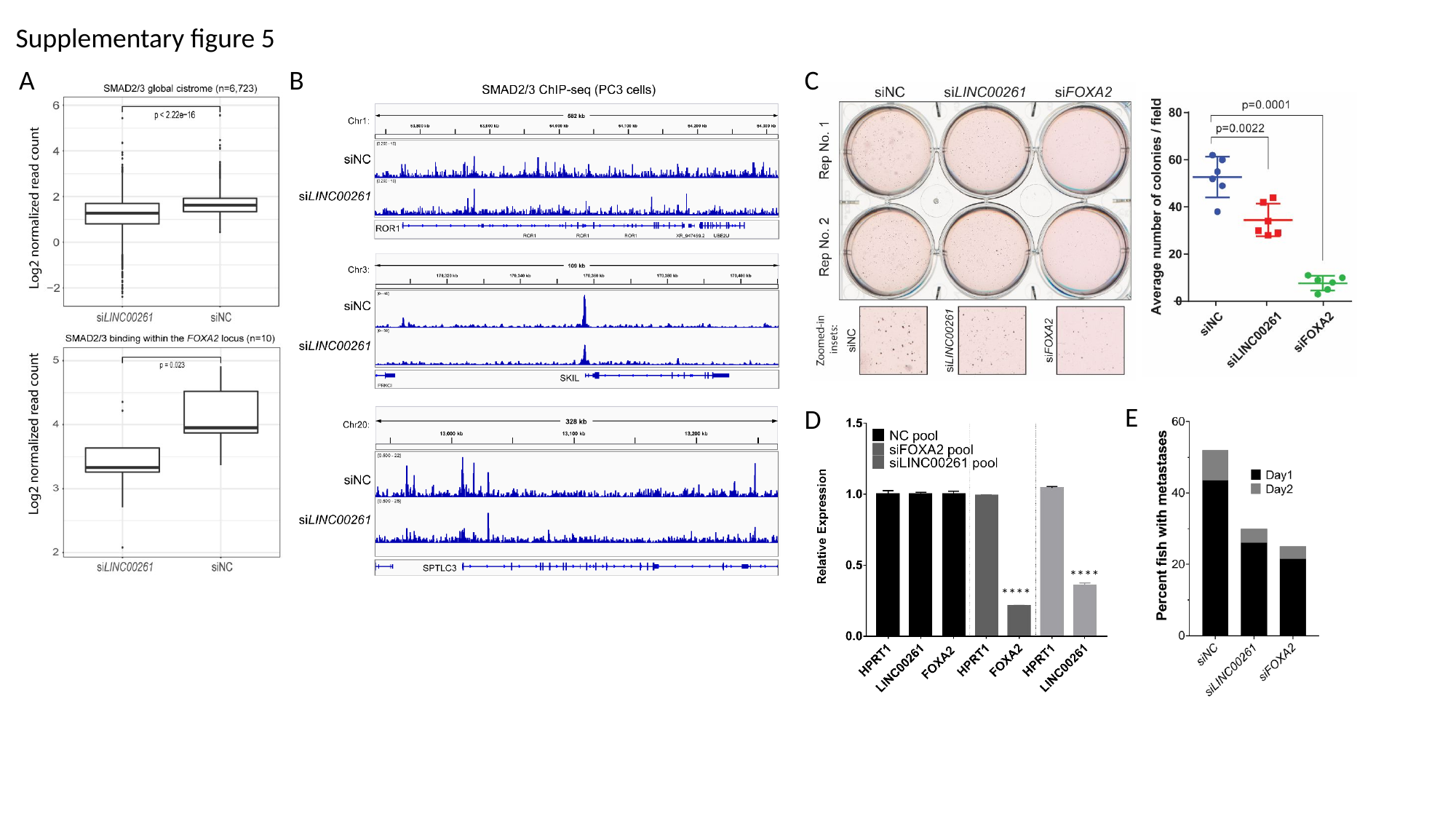

Supplementary figure 5
A
B
C
E
D
****
****

Supplement: Supplementary file 5 — Fig. S5. LINC00261 knockdown attenuates SMAD2/3 chromatin binding and hinders anchorage‐independent growth and metastatic ability of PC‐3 cells. (A) Top, Normalized SMAD2/3 ChIP‐seq read densities at all of its genomic binding sites (n = 6724) in PC‐3 cells or, bottom, cis‐regulatory genomic sites within the 4Mb window centered at the FOXA2 gene (n = 10). (B) SMAD2/3 ChIP‐seq read density tracks from siNC or siLINC00261‐treated PC‐3 cells at distinct genomic loci encoding bona fide TGF‐β1/SMAD target genes. (C) Left, Representative images of iodonitrotetrazolium chloride stained soft agar PC‐3 cell colonies after treatment with either the nontargeting, control siRNA (siNC) or siRNA targeting the LINC00261 (siLINC00261) transcript (two‐tailed t‐test). Insets at the bottom show magnified images of the colonies. Right, Average number of soft agar colonies per 10× field of siNC or siLINC00261‐treated PC‐3 cells. Distinct cells colonies were counted from three randomly chosen 10X field per well from two biological replicates. (D) Expression (qPCR) of LINC00261 and FOXA2 in siRNA‐treated PC‐3‐RFP cells used for the in zebrafish metastasis experiments. (E) Percentage of fish that show metastatic dissemination at 1 and 2 days after injection with PC‐3‐RFP cell treated with a nontargeting control (siControl; n = 52), siRNA targeting LINC00261 (n = 40), or siRNA targeting FOXA2 (n = 44). (A, D) was statistically analyzed using two‐tailed t‐test and (C) was analyzed using a one‐way ANOVA with Dunnett's post hoc test. [file MOL2-15-1921-s004.pptx]

## Slide 1
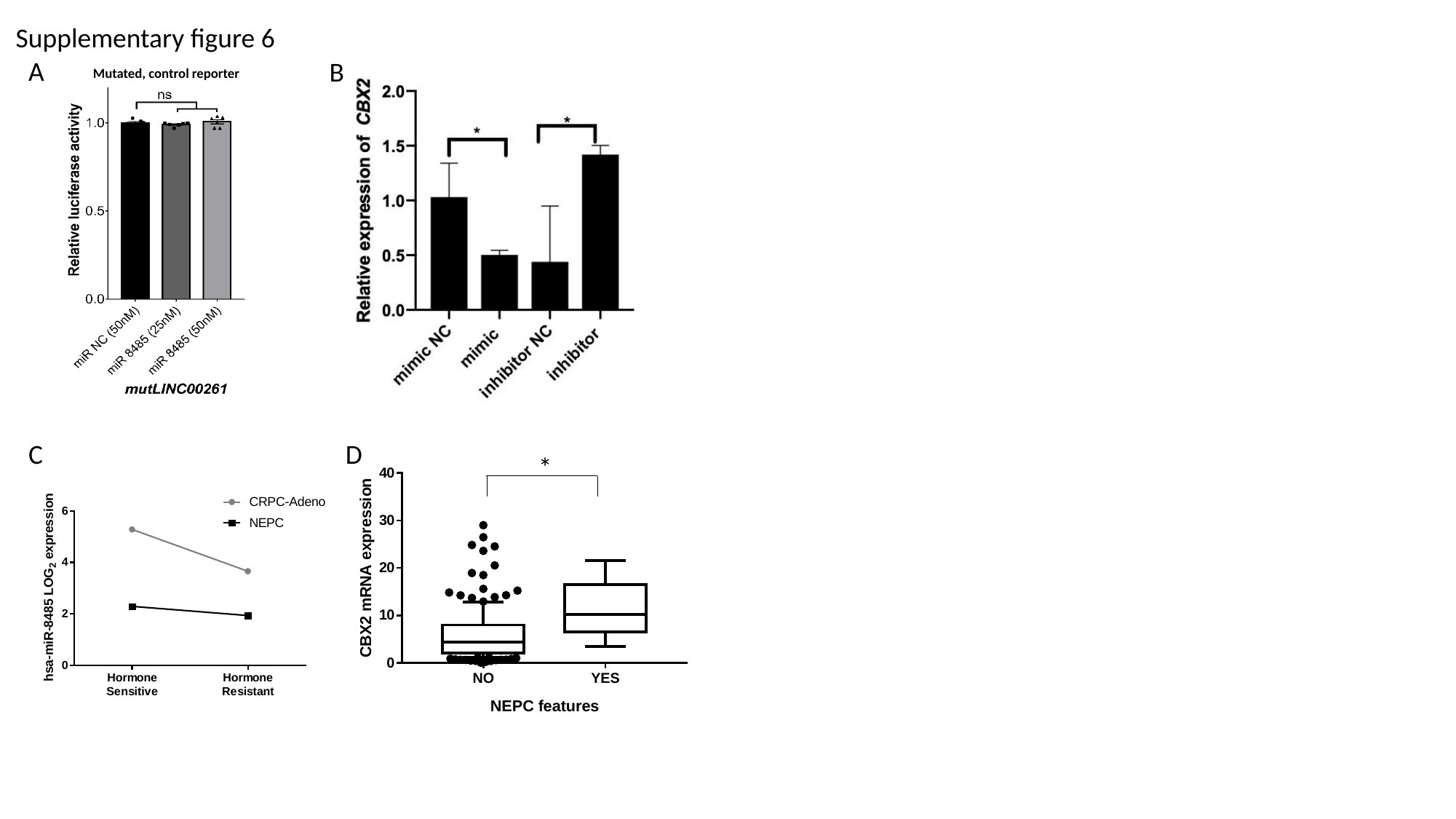

Supplementary figure 6
A
B
Mutated, control reporter
*
C
D

Supplement: Supplementary file 6 — Fig. S6. miR‐8485 mimic downregulates the expression of CBX2 to enable NEPC progression. (A) Luciferase activity from the control, mutated LINC00261 binding reporter assay (see Materials and methods) with transfection of miR‐8485 or a nontargeting control miRNA mimic in HEK293 cells. For each treatment group, reporter activity was normalized to background signals from the unmodified pmirGlo reporter alone. (B) Expression of CBX2 is significantly reduced upon expression of miR‐8485 mimic compared to mimic control. Expression of CBX2 is significantly increased upon miR‐8485 inhibition compared to inhibitor control. (C) Expression of has‐miR‐8485 in CRPC‐Adeno (n = 13) or NEPC (n = 4) patient‐derived PCa xenografts that are hormone‐sensitive or hormone‐resistant. (D) Expression of CBX2 in PCa patient samples with (n = 15) or without (n = 34) presentation of NEPC features. (B) Data analyzed by unpaired two‐tailed t‐test (*P < 0.05). [file MOL2-15-1921-s008.pptx]
