# Supplementary material for: The evolutionarily conserved long non‐coding RNA LINC00261 drives neuroendocrine prostate cancer proliferation and metastasis via distinct nuclear and cytoplasmic mechanisms
Source: Mol Oncol. 2021 Apr 26;15(7):1921–41. doi: 10.1002/1878-0261.12954 (PMC8253100; doi:10.1002/1878-0261.12954)
Supplement: Supplementary file 2 — Fig. S2. Subcellular localization of murine ortholog 9030622O22‐Rik and essentiality of LINC00261 in NEPC. (A) The expression of LINC00261's murine ortholog 9030622O22‐Rik in T23 (murine prostatic adenocarcinoma) and OPT7714 (murine NEPC). (B) Subcellular localization of Gapdh, Malat1, and 9030622O22‐Rik RNA transcripts in OPT7714 cells. (C) Expression of LINC00261 after treatment with LINC00261‐targeting siRNAs in PC‐3 cells (siRNA No. 1 P < 0.0001 siRNA No. 2 P < 0.0001, siRNA No. 3 P < 0.0001, all relative to siNC treated cells). (D) Representative images of PC‐3 cells 72 h after transfection with siNC or siLINC00261 (E) Viable PC‐3 cell counts after treatment with a control siRNA or three different siLINC00261 for 3 days. (F) Viable DU‐145 cells 7 days after treatment with negative control empty vector (NC) or LINC00261 overexpression vector (overexpression‐OE; **P = 0.0030). (A, B) data n = 3, ± SEM, Analyzed by graphpad prism 7 software. (C) Analyzed by one‐way ANOVA with Dunnett's post hoc test. (E) Analyzed by unpaired two‐tailed t‐test (n = 2). All data Analyzed using graphpad prism 7 software, n = 3 ± SEM unless otherwise stated. [file MOL2-15-1921-s003.pptx]

## Slide 1
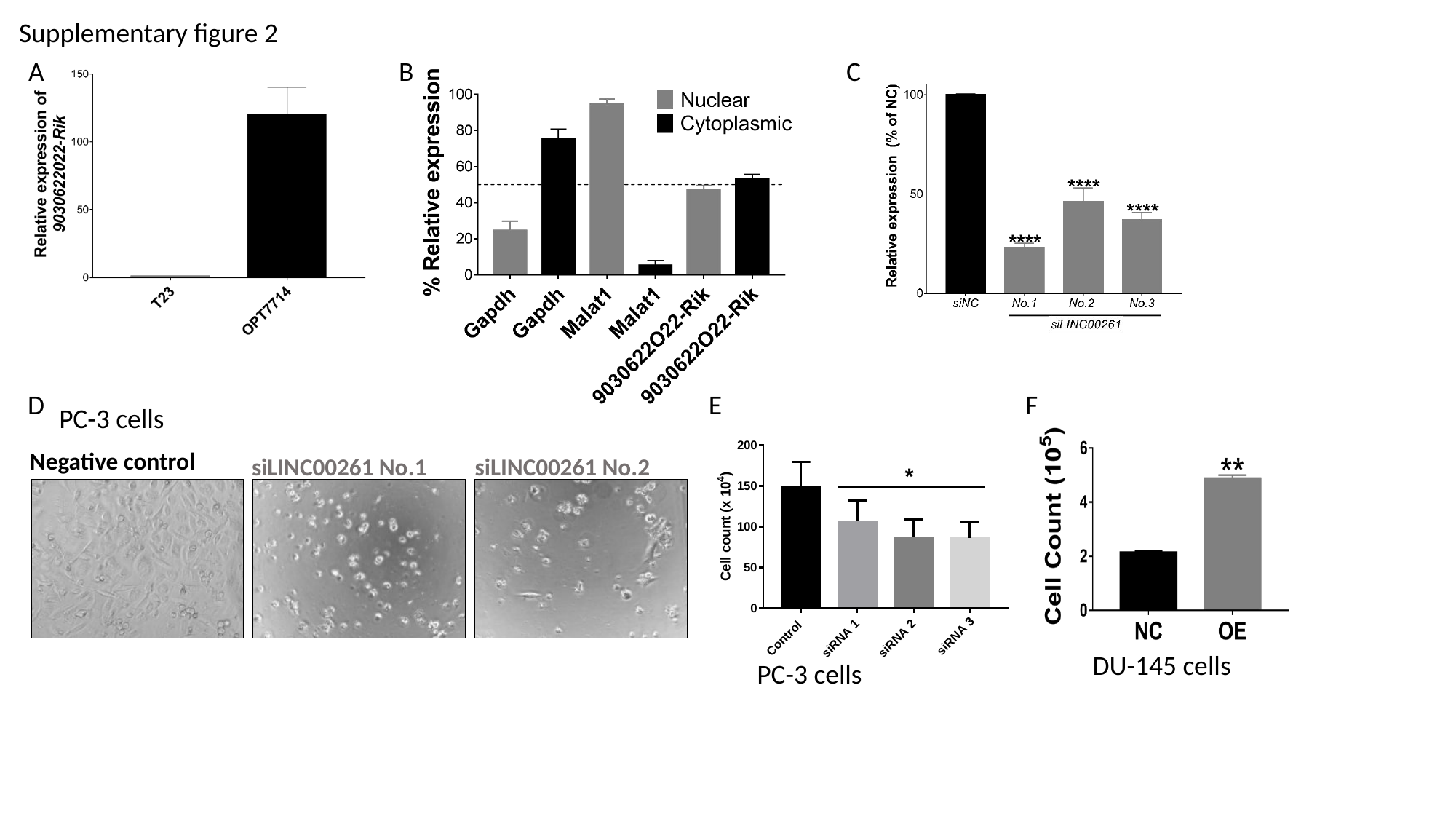

Supplementary figure 2
A
B
C
D
E
F
PC-3 cells
Negative control
siLINC00261 No.1
siLINC00261 No.2
DU-145 cells
PC-3 cells
