# Supplementary material for: The evolutionarily conserved long non‐coding RNA LINC00261 drives neuroendocrine prostate cancer proliferation and metastasis via distinct nuclear and cytoplasmic mechanisms
Source: Mol Oncol. 2021 Apr 26;15(7):1921–41. doi: 10.1002/1878-0261.12954 (PMC8253100; doi:10.1002/1878-0261.12954)
Supplement: Supplementary file 7 — Table S1. LINC00261 is associated with NEPC markers and FOXA2. Using co‐expression analysis on cBioPortal accessed from the Trento dataset LINC00261 expression has a tendency towards co‐occurrence with FOXA2, and the NEPC markers ENO2, CHGA, CBX5 and NCAM1 expression. There is also a tendency towards co‐occurrence between FOXA2 and the markers ENO2, CHGA, CBX5 and NCAM1. [file MOL2-15-1921-s005.pdf]

Supplementary Table 1: LINC00261 is associated with NEPC markers and FOXA2

Gene A	Gene B	p-Value	Log Odds Ratio	Association
LINC00261	FOXA2	<0.001	>3	Tendency towards co-occurrence
CHGA	FOXA2	<0.001	>3	Tendency towards co-occurrence
LINC00261	ENO2	<0.001	>3	Tendency towards co-occurrence
LINC00261	CHGA	<0.001	>3	Tendency towards co-occurrence
CHGA	ENO2	<0.001	>3	Tendency towards co-occurrence
ENO2	FOXA2	<0.001	>3	Tendency towards co-occurrence
LINC00261	CBX5	<0.001	>3	Tendency towards co-occurrence
ENO2	CBX5	<0.001	>3	Tendency towards co-occurrence
CHGA	NCAM1	0.002	>3	Tendency towards co-occurrence
NCAM1	FOXA2	0.002	>3	Tendency towards co-occurrence
CBX5	FOXA2	0.003	2.87	Tendency towards co-occurrence
ENO2	NCAM1	0.004	>3	Tendency towards co-occurrence
LINC00261	NCAM1	0.006	>3	Tendency towards co-occurrence
CHGA	CBX5	0.024	2.177	Tendency towards co-occurrence
