# Supplementary material for: The evolutionarily conserved long non‐coding RNA LINC00261 drives neuroendocrine prostate cancer proliferation and metastasis via distinct nuclear and cytoplasmic mechanisms
Source: Mol Oncol. 2021 Apr 26;15(7):1921–41. doi: 10.1002/1878-0261.12954 (PMC8253100; doi:10.1002/1878-0261.12954)
Supplement: Supplementary file 8 — Table S2. LINC00261 survival data from all other cancers available on the TANRIC database with the top correlated mRNA in each dataset. Using the TANRIC database each cancer type was queried for LINC00261 expression correlated with survival, and with mRNA. Only the top Spearman score mRNA is shown per dataset. Numbers of clinical samples in each data set is shown. [file MOL2-15-1921-s009.pdf]

*Supplementary Table 2: LINC00261 survival data from all other cancers available on the TANRIC database with the top correlated mRNA in each dataset.*

| Dataset name (number of samples) | Cox p-value | Top assocaited mRNA | Spearman correlation |
|---|---|---|---|
| Breast invasive carcinoma (837) | 0.711 | N/A | N/A |
| Bladder urothelial carcinoma (252) | 0.7304 | FOXA2 | 0.68 |
| Brain lower grade glioma (486) | 0.2382 | N/A | N/A |
| Cervical squamous cell carcinoma and endocervical adenocarcinoma (196) | 0.16474 | FOXA2 | 0.676 |
| Colon adenocarcinoma (157) | 0.79176 | GP2 | 0.611 |
| Glioblastoma multiforme (154) | 0.46798 | FOXA2 | 0.753 |
| Head and neck squamous cell carcinoma (426) | 0.0062936 | N/A | N/A |
| Kidney chromophobe (66) | N/A | N/A | N/A |
| Kidney renal cell carcinoma (448) | 0.00057578 | FOXA2 | 0.564 |
| Kidney papillary cell carcinoma (198) | 0.031107 | HERC2P4 | 0.496 |
| Liver hepatocellular carcinoma (200) | 0.63711 | ACAD11 | 0.778 |
| Lung adenocarcinoma (488) | 0.10005 | FOXA2 | 0.91 |
| Lung squamous cell carcinoma (220) | 0.67554 | FOXA2 | 0.876 |
| Ovarian serous adenocarcinoma (412) | 0.23552 | FOXA2 | 0.843 |
| Rectum adenocarcinoma (71) | 0.60682 | MIA3 | 0.743 |
| Skin cutaneous melanoma (226) | N/A | N/A | N/A |
| Stomach adenocarcinoma (285) | 0.38887 | FOXA2 | 0.756 |
| Thyroid carcinoma (497) | 0.036432 | FOXA2 | 0.848 |
| Uterine corpus endometroid carcinoma (316) | 0.18807 | FOXA2 | 0.904 |
