# Supplementary material for: The evolutionarily conserved long non‐coding RNA LINC00261 drives neuroendocrine prostate cancer proliferation and metastasis via distinct nuclear and cytoplasmic mechanisms
Source: Mol Oncol. 2021 Apr 26;15(7):1921–41. doi: 10.1002/1878-0261.12954 (PMC8253100; doi:10.1002/1878-0261.12954)
Supplement: Supplementary file 9 — Table S3. List of differentially expressed genes (RNA‐seq) in siLINC00261‐treated PC‐3 cells. RNA‐seq analysis was carried out using criteria described in Materials and methods section. In total, the analyses revealed 180 down‐regulated transcripts, and 519 up‐regulated transcripts upon LINC00261 knock‐down. [file MOL2-15-1921-s002.pdf]

Supplementary Table 3: List of differentially expressed genes (RNASeq) in si*LINC00261* -treated PC-3 cells

ID	siRNA Avg (log2)	NC Avg (log2)	Fold Change	P-val	FDR P-val	Gene Symbol
LINC00261	1.53	4.53	-7.99	0.0027	0.0677	LINC00261
ACO1	3.62	6.11	-5.61	0.0004	0.0319	ACO1
TOR1B	2.97	5.18	-4.63	0.0006	0.0366	TOR1B
MDH1	6.5	8.55	-4.12	0.0008	0.0394	MDH1
GPR98	2.49	4.51	-4.06	0.0017	0.0571	GPR98
HNRNPAB	7.41	9.32	-3.75	1.21E-05	0.0121	HNRNPAB
PTMA	9.71	11.56	-3.62	0.0004	0.0317	PTMA
ADD3	2.54	4.38	-3.58	0.0045	0.0839	ADD3
AXL	4.81	6.59	-3.43	0.0002	0.0278	AXL
HIST1H4A	5.08	6.85	-3.39	0.0033	0.0734	HIST1H4A
MAD2L1	5.03	6.78	-3.38	0.0017	0.0571	MAD2L1
FABP4	1.64	3.39	-3.37	0.0029	0.0706	FABP4
PADI2	0.1	1.82	-3.28	0.0059	0.0961	PADI2
SLC4A7	5.78	7.48	-3.24	0.0038	0.0785	SLC4A7
PALMD	1.88	3.56	-3.19	0.0032	0.0728	PALMD
TTC3P1	1.26	2.93	-3.18	0.0031	0.0721	TTC3P1
PKI55	2.83	4.49	-3.14	0.006	0.0967	PKI55
ELOVL5	6.62	8.24	-3.08	1.52E-05	0.0126	ELOVL5
PRKDC	6.68	8.28	-3.02	1.59E-05	0.0126	PRKDC
HIST1H3B	7.12	8.68	-2.94	0.004	0.0796	HIST1H3B
C20orf151	1.05	2.6	-2.92	0.0337	0.2208	C20orf151
MLF1IP	3.85	5.38	-2.87	0.0021	0.0626	MLF1IP
FLJ22184	1.96	3.48	-2.85	0.0008	0.0402	FLJ22184
ODC1	9.41	10.9	-2.8	0.0003	0.0293	ODC1
MYH15	1.89	3.38	-2.8	0.0237	0.1863	MYH15
NT5DC2	5.79	7.27	-2.79	7.39E-06	0.0104	NT5DC2
DANCR	5.7	7.18	-2.79	9.30E-05	0.0223	DANCR
CALB2	0.48	1.95	-2.78	0.0189	0.165	CALB2
MBNL3	1.56	3.01	-2.73	0.0038	0.0786	MBNL3
TULP4	2.75	4.2	-2.73	0.0288	0.2039	TULP4
CROT	3.56	5	-2.72	6.87E-05	0.0214	CROT
BRMS1L	3.49	4.92	-2.7	0.0031	0.0718	BRMS1L
HMGA2	6.17	7.57	-2.66	0.0007	0.039	HMGA2
SHH	1.58	2.96	-2.61	0.0045	0.0839	SHH
TTC3	5.66	7.05	-2.61	0.0204	0.1721	TTC3
EPN3	2.17	3.55	-2.6	0.0006	0.0377	EPN3
CYFIP2	2.79	4.17	-2.59	0.0012	0.05	CYFIP2
ANKRD36BP1	5.62	6.99	-2.58	0.0006	0.0366	ANKRD36BP1
VASH2	1.41	2.78	-2.58	0.0048	0.0871	VASH2
MAP7D2	1.41	2.77	-2.56	0.0001	0.0253	MAP7D2
SAMD5	1.61	2.97	-2.56	0.035	0.225	SAMD5
HELLS	4.27	5.62	-2.55	0.0015	0.0537	HELLS

POLR1A	5.47	6.82	-2.55	0.0193	0.1672	POLR1A
DPYSL3	4.32	5.65	-2.52	0.0006	0.0373	DPYSL3
EPRS	6.79	8.11	-2.5	0.0009	0.0428	EPRS
PHOSPHO1	0.32	1.64	-2.5	0.0161	0.1526	PHOSPHO1
BZW1	8.55	9.86	-2.48	8.66E-05	0.0222	BZW1
HIST1H4B	6.29	7.6	-2.48	0.0004	0.0339	HIST1H4B
MT1F	3.43	4.73	-2.47	0.0038	0.0786	MT1F
ERLIN2	3.92	5.22	-2.47	0.0043	0.0819	ERLIN2
ANKRD22	2.07	3.37	-2.46	0.0132	0.1406	ANKRD22
SCML1	0.8	2.09	-2.46	0.0186	0.1634	SCML1
VCAN	2.69	3.98	-2.46	0.0471	0.2653	VCAN
ABCA13	1.84	3.13	-2.45	0.0039	0.0792	ABCA13
SFT2D2	5.27	6.56	-2.45	0.0055	0.0927	SFT2D2
IDNK	2.3	3.6	-2.45	0.0061	0.0973	IDNK
AIF1L	3	4.29	-2.45	0.0155	0.1513	AIF1L
MYH9	8.38	9.67	-2.44	0.0011	0.0476	MYH9
RAB31	3.5	4.78	-2.43	0.0036	0.0773	RAB31
PIGH	6.6	7.86	-2.41	0.0008	0.0394	PIGH
PPARA	3.4	4.66	-2.4	0.0043	0.0825	PPARA
IQCG	2.02	3.29	-2.4	0.0088	0.1158	IQCG
CYB5A	4.14	5.4	-2.39	0.0013	0.0519	CYB5A
ASPM	4.8	6.06	-2.39	0.0059	0.0961	ASPM
SH3YL1	3.13	4.38	-2.38	0.0031	0.0721	SH3YL1
ALDH1A3	6.83	8.08	-2.38	0.0032	0.0728	ALDH1A3
PRR11	5.93	7.18	-2.37	0.0006	0.0366	PRR11
SLC26A2	4.21	5.45	-2.37	0.0107	0.1272	SLC26A2
PLK1	5.49	6.73	-2.36	0.0194	0.1676	PLK1
APOA1BP	5.79	7.02	-2.35	0.0015	0.0537	APOA1BP
CHML	4.91	6.14	-2.35	0.0243	0.1888	CHML
MYH10	4.69	5.91	-2.33	7.89E-05	0.0219	MYH10
KIF5C	1.74	2.97	-2.33	0.0006	0.0366	KIF5C
SLC25A43	3.6	4.82	-2.33	0.0017	0.0561	SLC25A43
ESM1	3.98	5.2	-2.33	0.0205	0.1728	ESM1
SCD	6.92	8.14	-2.33	0.0262	0.1954	SCD
RNF125	1.47	2.69	-2.33	0.0399	0.243	RNF125
PDE5A	3.81	5.03	-2.32	0.0001	0.0253	PDE5A
LCP1	6.61	7.82	-2.32	0.0037	0.0779	LCP1
FAM5B	2.27	3.48	-2.32	0.0172	0.158	FAM5B
HIST1H4H	7.79	9	-2.31	0.0024	0.0653	HIST1H4H
RPL3	8.94	10.15	-2.31	0.0158	0.1524	RPL3
ARG2	2.81	4.01	-2.3	0.0032	0.0724	ARG2
DNAJC12	3.13	4.33	-2.29	0.0062	0.0977	DNAJC12
PRKCA	5.82	7.01	-2.29	0.008	0.1107	PRKCA
CIT	4.65	5.84	-2.28	0.0088	0.1156	CIT
CBX2	4.07	5.26	-2.28	0.014	0.1435	CBX2
RASSF3	4.51	5.7	-2.27	0.0056	0.094	RASSF3
RPS10	9.29	10.47	-2.27	0.0088	0.1156	RPS10

HTR1D	1.39	2.57	-2.27	0.0091	0.1171	HTR1D
INTS1	4.81	5.99	-2.26	0.016	0.1525	INTS1
SMN2	3.64	4.81	-2.25	0.0005	0.0352	SMN2
PITRM1	4.7	5.86	-2.24	0.0005	0.035	PITRM1
MKI67	5.69	6.85	-2.23	0.0062	0.0975	MKI67
GCAT	2.1	3.26	-2.23	0.0062	0.0978	GCAT
DDX39A	6.81	7.97	-2.23	0.0153	0.1499	DDX39A
SARDH	1.92	3.08	-2.23	0.0277	0.1999	SARDH
ISOC1	3.53	4.68	-2.22	0.0007	0.039	ISOC1
TXNDC16	4.54	5.69	-2.22	0.0014	0.0535	TXNDC16
PYGL	7.11	8.26	-2.22	0.0064	0.0989	PYGL
MMAB	4.29	5.44	-2.22	0.0138	0.1428	MMAB
TMEM52	1.84	2.99	-2.21	0.0087	0.1154	TMEM52
RDH10	6.26	7.4	-2.21	0.0096	0.121	RDH10
RCOR2	1.28	2.42	-2.2	0.0004	0.0311	RCOR2
MLEC	6.83	7.97	-2.2	0.0006	0.0366	MLEC
HIST1H1D	6.64	7.78	-2.2	0.0008	0.04	HIST1H1D
LOC100506668	2.82	3.96	-2.2	0.0027	0.0677	LOC100506668
PTGFRN	3.38	4.52	-2.2	0.014	0.1435	PTGFRN
HIST1H2BH	4.43	5.57	-2.19	0.0054	0.0921	HIST1H2BH
CRISPLD2	1.1	2.23	-2.19	0.0129	0.1395	CRISPLD2
UNC119B	5.15	6.27	-2.18	0.0002	0.0254	UNC119B
SYT16	4.75	5.87	-2.18	0.0005	0.0342	SYT16
DENR	6.17	7.29	-2.18	0.0006	0.0366	DENR
SYTL1	1.82	2.95	-2.18	0.0119	0.1349	SYTL1
RAB11FIP4	1.3	2.43	-2.18	0.021	0.1748	RAB11FIP4
DLGAP5	6.91	8.03	-2.17	0.0025	0.0662	DLGAP5
SAPCD2	3.41	4.52	-2.17	0.0033	0.0739	SAPCD2
HIST1H2AI	5.73	6.85	-2.17	0.0049	0.0871	HIST1H2AI
TFRC	9.29	10.4	-2.16	0.0009	0.0416	TFRC
CDCA7L	5.41	6.52	-2.15	0.0022	0.0628	CDCA7L
TUBB4A	4.14	5.24	-2.15	0.017	0.1576	TUBB4A
LRRC34	2.59	3.69	-2.15	0.0221	0.1794	LRRC34
MT1X	6.3	7.4	-2.14	0.0092	0.1176	MT1X
HIST1H4E	6.36	7.46	-2.14	0.0137	0.1425	HIST1H4E
DNAAF3	4.46	5.55	-2.13	0.0001	0.0253	DNAAF3
MPND	1.21	2.3	-2.13	0.0466	0.2641	MPND
ODF2	2.39	3.47	-2.12	0.0189	0.1649	ODF2
HEATR1	5.93	7	-2.11	0.0019	0.0584	HEATR1
LOC100128881	3.86	4.94	-2.11	0.0022	0.0628	LOC100128881
PSRC1	2.14	3.22	-2.11	0.0029	0.0706	PSRC1
PALM3	2.43	3.5	-2.11	0.0303	0.2075	PALM3
HNRNPH3	6.38	7.45	-2.1	0.0021	0.0616	HNRNPH3
SETD7	5.57	6.64	-2.1	0.0022	0.0628	SETD7
HIST1H2BD	7.8	8.87	-2.1	0.0029	0.0704	HIST1H2BD
ERVMER34-1	1.95	3.02	-2.1	0.0154	0.1502	ERVMER34-1
LOC92249	3.91	4.99	-2.1	0.0364	0.2311	LOC92249

PTPN14	4.89	5.95	-2.09	0.0006	0.0377	PTPN14
CDC20	5.2	6.26	-2.09	0.0007	0.0383	CDC20
CTH	2.98	4.05	-2.09	0.0008	0.0412	CTH
RRAS2	4.25	5.31	-2.08	0.0007	0.0388	RRAS2
AKAP12	7.25	8.31	-2.08	0.0104	0.1261	AKAP12
ZNF488	1.1	2.16	-2.08	0.0198	0.1691	ZNF488
EIF2C1	4.94	5.99	-2.07	0.0003	0.03	EIF2C1
C7orf49	4.28	5.33	-2.07	0.001	0.0443	C7orf49
PTPN18	2.5	3.55	-2.07	0.0014	0.0535	PTPN18
POLA1	4.71	5.76	-2.07	0.0095	0.1198	POLA1
PABPC4	8.16	9.21	-2.07	0.0139	0.143	PABPC4
GATSL2	1.44	2.49	-2.07	0.0177	0.1599	GATSL2
HIST1H1E	6.72	7.77	-2.07	0.0199	0.1701	HIST1H1E
EEF1G	9.99	11.04	-2.07	0.0275	0.1993	EEF1G
BSPRY	2.24	3.29	-2.07	0.0351	0.2257	BSPRY
FUT1	1.62	2.66	-2.06	0.0035	0.0761	FUT1
DYNC2H1	3.84	4.88	-2.06	0.0196	0.1686	DYNC2H1
CACYBP	5.42	6.45	-2.05	0.0072	0.1046	CACYBP
EIF4B	7.57	8.6	-2.05	0.0269	0.1978	EIF4B
NELF	4.87	5.91	-2.05	0.027	0.1979	NELF
HNRNPA0	4.25	5.28	-2.04	0.0003	0.0293	HNRNPA0
DCAF13	6.84	7.87	-2.04	0.0005	0.0352	DCAF13
PTPLB	6.18	7.21	-2.04	0.0018	0.0583	PTPLB
DEPDC1	4.24	5.27	-2.04	0.0048	0.0869	DEPDC1
SLC16A2	2.57	3.6	-2.04	0.0234	0.1847	SLC16A2
HIST1H4C	7.81	8.83	-2.03	0.0001	0.0253	HIST1H4C
LOC100288842	2.59	3.61	-2.03	0.0006	0.0366	LOC100288842
SMN1	4.94	5.96	-2.03	0.001	0.0438	SMN1
KIAA1462	5.19	6.21	-2.03	0.0027	0.0683	KIAA1462
CCNB1IP1	5.3	6.31	-2.02	0.0008	0.0412	CCNB1IP1
LOC730101	3.6	4.61	-2.02	0.0012	0.0492	LOC730101
REPS2	2.71	3.72	-2.02	0.0017	0.0561	REPS2
IGSF9	2.45	3.46	-2.02	0.0026	0.0676	IGSF9
PUS1	3.16	4.18	-2.02	0.0044	0.0838	PUS1
SMPD2	2.02	3.03	-2.02	0.0115	0.1319	SMPD2
RPL19	9.14	10.16	-2.02	0.0295	0.2058	RPL19
CCDC113	2.69	3.7	-2.02	0.0448	0.2587	CCDC113
FAM18B1	4.76	5.77	-2.01	0.0037	0.0776	FAM18B1
COMMD8	3.65	4.66	-2.01	0.0084	0.1137	COMMD8
DFFA	5.71	6.71	-2.01	0.0106	0.1271	DFFA
PPAPDC1B	3.84	4.85	-2.01	0.048	0.2685	PPAPDC1B
DESI2	4.78	5.78	-2	0.0108	0.1277	DESI2
RALGAPA2	4.18	5.18	-2	0.0172	0.158	RALGAPA2
EXPH5	4.58	5.59	-2	0.0298	0.2061	EXPH5
PELI1	4.93	3.93	2	0.0015	0.0537	PELI1
KLHL21	5.98	4.98	2	0.0084	0.1132	KLHL21
FAM214B	3.73	2.73	2	0.0208	0.1739	FAM214B

PDE8B	1.23	0.23	2	0.0247	0.1904	PDE8B
CPEB3	2.93	1.93	2	0.0333	0.2202	CPEB3
TMEM50A	6.88	5.88	2.01	0.0007	0.0379	TMEM50A
C5orf30	5	3.99	2.01	0.0023	0.0644	C5orf30
FAM122C	1.56	0.55	2.01	0.0024	0.066	FAM122C
ZNRF2	5.11	4.11	2.01	0.0029	0.0704	ZNRF2
APCDD1L	1.82	0.81	2.01	0.0069	0.1025	APCDD1L
CXorf49	1.15	0.14	2.01	0.0073	0.1058	CXorf49
TBX1	2.15	1.14	2.01	0.0087	0.1148	TBX1
SERPINB1	5.9	4.9	2.01	0.0184	0.1629	SERPINB1
RAPGEF3	2.38	1.36	2.01	0.0382	0.2372	RAPGEF3
SPRY2	7.35	6.33	2.02	0.0001	0.0235	SPRY2
IRF2	5.9	4.89	2.02	0.0005	0.0362	IRF2
CMTM7	6.18	5.17	2.02	0.0014	0.0535	CMTM7
TNIP1	8.37	7.35	2.02	0.0038	0.0786	TNIP1
ABCA1	5.13	4.12	2.02	0.0061	0.0971	ABCA1
SLFN5	6.99	5.98	2.02	0.011	0.1287	SLFN5
RNF19B	7.12	6.1	2.03	0.0006	0.0378	RNF19B
C16orf70	6.34	5.32	2.03	0.0007	0.0394	C16orf70
TNFRSF1B	1.88	0.86	2.03	0.0045	0.0838	TNFRSF1B
SOX9	6.81	5.79	2.03	0.0077	0.1087	SOX9
ONECUT3	3.78	2.76	2.03	0.0253	0.192	ONECUT3
TINAGL1	6.65	5.62	2.04	0.0025	0.0662	TINAGL1
PKD1	4.12	3.09	2.04	0.0025	0.0662	PKD1
PHACTR2	3.66	2.63	2.04	0.0048	0.0869	PHACTR2
MOV10	6.04	5.01	2.04	0.0069	0.1026	MOV10
GLRX	6.74	5.71	2.04	0.0222	0.1794	GLRX
FLI1	2.97	1.93	2.05	0.0031	0.0719	FLI1
RDH11	8.04	7	2.05	0.0032	0.0729	RDH11
MXD1	5.19	4.15	2.05	0.005	0.0879	MXD1
POLR3G	5.44	4.41	2.05	0.0054	0.0913	POLR3G
SQRDL	5.91	4.87	2.05	0.0323	0.2158	SQRDL
MCL1	8	6.96	2.06	0.0012	0.0492	MCL1
TNFRSF14	2.98	1.94	2.06	0.0017	0.0561	TNFRSF14
MTF2	5.66	4.62	2.06	0.0028	0.069	MTF2
CLSTN1	7.56	6.52	2.06	0.008	0.1107	CLSTN1
SERPINB7	1.67	0.63	2.06	0.0313	0.2113	SERPINB7
ATXN7L3B	7.75	6.7	2.07	8.37E-05	0.0222	ATXN7L3B
APAF1	4.35	3.3	2.07	0.0008	0.0404	APAF1
FUT4	3.12	2.07	2.07	0.0018	0.0573	FUT4
APPBP2	7.42	6.37	2.07	0.0027	0.0679	APPBP2
XDH	2.06	1.02	2.07	0.004	0.0795	XDH
IGFBP4	9.22	8.18	2.07	0.0085	0.1139	IGFBP4
SPHK1	6.93	5.88	2.07	0.0106	0.1271	SPHK1
OR52K2	1.05	0	2.07	0.016	0.1525	OR52K2
RASA2	4.81	3.75	2.08	0.0005	0.0352	RASA2
FAM26E	1.19	0.13	2.08	0.0007	0.0383	FAM26E

SMOX	7.66	6.61	2.08	0.003	0.0712	SMOX
CASP4	7.87	6.82	2.08	0.0055	0.0928	CASP4
PCDHGB5	4.08	3.03	2.08	0.0156	0.1517	PCDHGB5
FN1	5.18	4.13	2.08	0.0223	0.1796	FN1
PTPRH	3.93	2.87	2.09	0.0032	0.0725	PTPRH
ERAP2	6.1	5.04	2.09	0.0081	0.1113	ERAP2
TOR2A	3.86	2.8	2.09	0.0111	0.1291	TOR2A
MORF4L1	4.27	3.21	2.09	0.0111	0.1294	MORF4L1
CAPN3	1.71	0.64	2.1	0.0003	0.0293	CAPN3
ROCK2	7.81	6.75	2.1	0.0007	0.039	ROCK2
PDGFA	5.93	4.86	2.1	0.0011	0.0468	PDGFA
LOC645212	4.11	3.03	2.1	0.0059	0.0961	LOC645212
WARS	7.62	6.55	2.1	0.0148	0.148	WARS
ERMAP	3.22	2.15	2.11	0.0001	0.0253	ERMAP
ITFG3	6.34	5.27	2.11	0.0036	0.0775	ITFG3
SGCB	7.81	6.73	2.11	0.0102	0.1249	SGCB
PTK2B	4.73	3.65	2.11	0.0122	0.1367	PTK2B
SRGN	9.75	8.67	2.12	7.19E-05	0.0214	SRGN
NOD1	4.52	3.44	2.12	0.0005	0.0364	NOD1
COQ10A	3.29	2.2	2.12	0.0011	0.0474	COQ10A
C1QL1	3.59	2.51	2.12	0.0024	0.0647	C1QL1
TACSTD2	6.88	5.79	2.12	0.0074	0.1066	TACSTD2
TP53INP1	1.93	0.85	2.12	0.011	0.1288	TP53INP1
PRKCE	4.12	3.04	2.12	0.0126	0.1384	PRKCE
AURKC	1.94	0.85	2.12	0.0337	0.2208	AURKC
CELSR3	2.33	1.24	2.12	0.0399	0.2429	CELSR3
CSGALNACT1	1.33	0.24	2.13	7.87E-05	0.0219	CSGALNACT1
BTN3A1	4.96	3.88	2.13	0.0002	0.0257	BTN3A1
PATL1	7.93	6.84	2.13	0.0005	0.0363	PATL1
PRDM1	3.13	2.04	2.13	0.0045	0.0838	PRDM1
KLF4	5.28	4.19	2.13	0.0082	0.1121	KLF4
CDKN1A	6.08	4.99	2.13	0.0398	0.2427	CDKN1A
WDFY1	6.56	5.46	2.14	4.08E-06	0.0095	WDFY1
SELL	1.77	0.67	2.14	0.0027	0.0686	SELL
THAP10	3.53	2.43	2.14	0.0033	0.0739	THAP10
LOC100505633	2.23	1.13	2.14	0.0039	0.0792	LOC100505633
BACH2	3.27	2.17	2.14	0.0178	0.1603	BACH2
MST4	5.71	4.61	2.14	0.023	0.1831	MST4
CLTA	1.57	0.47	2.15	0.0013	0.0517	CLTA
S100A3	2.98	1.87	2.15	0.0023	0.0636	S100A3
DUSP10	4.94	3.84	2.15	0.0047	0.0861	DUSP10
DNAJB6	8.13	7.03	2.15	0.0048	0.087	DNAJB6
C14orf159	1.1	0	2.15	0.0061	0.0973	C14orf159
PLS3	9.8	8.69	2.16	0.0006	0.0366	PLS3
PMP22	5.83	4.73	2.16	0.0006	0.0377	PMP22
WASH5P	3.05	1.93	2.16	0.0027	0.068	WASH5P
CACNG6	1.11	0	2.16	0.0029	0.0704	CACNG6

IL28RA	3.61	2.5	2.16	0.0067	0.1015	IL28RA
TNFRSF6B	3.26	2.14	2.16	0.011	0.129	TNFRSF6B
CCNA1	3.17	2.05	2.17	0.0014	0.0527	CCNA1
CASP7	2.02	0.9	2.17	0.0019	0.0584	CASP7
SPOPL	4.94	3.83	2.17	0.0032	0.073	SPOPL
FOXC1	3.67	2.55	2.17	0.0035	0.0756	FOXC1
BAK1	6.68	5.56	2.17	0.005	0.0876	BAK1
SPTSSB	6.84	5.72	2.17	0.0174	0.1586	SPTSSB
ELAVL2	3.09	1.96	2.18	6.62E-06	0.0104	ELAVL2
CRIM1	8.02	6.89	2.18	0.0004	0.0336	CRIM1
ZHX2	5.66	4.54	2.18	0.0052	0.0899	ZHX2
CHSY1	5.77	4.64	2.18	0.0083	0.1129	CHSY1
CHMP5	8.19	7.06	2.19	0.0002	0.0276	CHMP5
MMD	5.04	3.91	2.19	0.0006	0.0366	MMD
RSL24D1	7.8	6.68	2.19	0.0008	0.0408	RSL24D1
CCDC68	4.24	3.11	2.19	0.0049	0.0872	CCDC68
CRYZ	6.5	5.37	2.19	0.0056	0.094	CRYZ
ATXN7	1.77	0.65	2.19	0.0108	0.1277	ATXN7
HIST3H2BB	6.62	5.49	2.19	0.0135	0.142	HIST3H2BB
PATL2	1.61	0.48	2.19	0.0202	0.1714	PATL2
SEMA3B	4.03	2.9	2.19	0.0472	0.2655	SEMA3B
CCND3	6.41	5.28	2.2	0.0006	0.0366	CCND3
PDE4B	3.77	2.63	2.2	0.0012	0.0498	PDE4B
RASD1	4.06	2.92	2.2	0.0039	0.0793	RASD1
CD7	2.75	1.61	2.2	0.0059	0.0961	CD7
GRAMD3	6.26	5.12	2.2	0.0124	0.1375	GRAMD3
KIAA0913	7.56	6.42	2.21	0.0005	0.0353	KIAA0913
TRAF5	3.1	1.96	2.21	0.0014	0.0525	TRAF5
F3	3.24	2.09	2.21	0.0019	0.0584	F3
SOD2	9.37	8.23	2.21	0.0073	0.1063	SOD2
NEK8	1.94	0.8	2.21	0.0152	0.1496	NEK8
XKR8	5.75	4.6	2.22	0.0001	0.0253	XKR8
SIRPB1	2.67	1.53	2.22	0.0018	0.0573	SIRPB1
THG1L	6.36	5.21	2.22	0.0026	0.0672	THG1L
PTN	2.68	1.53	2.22	0.008	0.111	PTN
GPR153	7.15	6	2.22	0.0183	0.1623	GPR153
PRNP	4.09	2.94	2.22	0.0192	0.1661	PRNP
CLDN23	2.11	0.96	2.22	0.0249	0.1911	CLDN23
FAM105A	5.57	4.41	2.23	0.005	0.0879	FAM105A
IL1B	7.85	6.69	2.23	0.0053	0.0908	IL1B
GATA6	4.91	3.75	2.23	0.0078	0.1097	GATA6
PPIC	4.76	3.6	2.24	0.0005	0.0342	PPIC
TAF8	4.53	3.36	2.25	0.0004	0.0319	TAF8
CD58	6.22	5.05	2.25	0.01	0.1234	CD58
DLG4	4.69	3.52	2.25	0.0262	0.1954	DLG4
ADARB1	5.76	4.58	2.26	7.15E-05	0.0214	ADARB1
TGFBR3	4.45	3.28	2.26	0.0023	0.0636	TGFBR3

ACP2	5.5	4.32	2.26	0.0051	0.089	ACP2
LAP3	7.53	6.35	2.26	0.0093	0.1182	LAP3
ULBP2	5.08	3.9	2.26	0.0104	0.126	ULBP2
LMBRD1	2.82	1.65	2.26	0.0238	0.1867	LMBRD1
KIAA0355	3.5	2.32	2.26	0.0338	0.2208	KIAA0355
CSAG1	1.71	0.53	2.26	0.0436	0.2548	CSAG1
LRRC58	5.92	4.73	2.27	0.0018	0.0581	LRRC58
ATXN7L1	4.93	3.75	2.27	0.0019	0.0592	ATXN7L1
DUSP6	6.55	5.37	2.27	0.0091	0.117	DUSP6
LEPROT	6.84	5.66	2.27	0.0092	0.1176	LEPROT
GPR137C	2.67	1.49	2.27	0.0254	0.1923	GPR137C
SUCO	8.54	7.35	2.28	0.0002	0.0278	SUCO
BTN3A3	5.4	4.21	2.28	0.001	0.0444	BTN3A3
CXCL3	6.52	5.33	2.28	0.0049	0.0873	CXCL3
LOC728613	4.35	3.17	2.28	0.0179	0.1608	LOC728613
SUSD3	4.81	3.62	2.29	0.0015	0.0547	SUSD3
PLA2G4C	1.59	0.39	2.29	0.0019	0.0586	PLA2G4C
HLA-A	8.28	7.08	2.29	0.0031	0.0719	HLA-A
PSMB8	6.34	5.14	2.29	0.0035	0.076	PSMB8
PDK4	3.23	2.03	2.29	0.0042	0.0811	PDK4
RPS6KC1	6.79	5.59	2.29	0.0393	0.2408	RPS6KC1
INHBA	8.53	7.32	2.3	0.0024	0.0649	INHBA
MAP2	2.11	0.91	2.3	0.0134	0.1417	MAP2
IRF9	6.04	4.83	2.31	0.0008	0.0399	IRF9
LOC100506710	5.43	4.22	2.31	0.0029	0.0702	LOC100506710
PCDH1	3.6	2.38	2.32	0.0015	0.0537	PCDH1
CDC7	4.54	3.33	2.32	0.0102	0.1248	CDC7
NDUFA5	6.65	5.43	2.33	0.0034	0.0746	NDUFA5
DAPP1	4.09	2.87	2.33	0.0069	0.1026	DAPP1
DUSP1	8.22	7	2.34	0.0026	0.0671	DUSP1
LIF	6.96	5.73	2.34	0.0099	0.1223	LIF
PCDHB2	4.39	3.16	2.34	0.0179	0.1605	PCDHB2
MBNL2	6.46	5.23	2.35	0.0151	0.1495	MBNL2
HAS3	5.4	4.16	2.35	0.0152	0.1497	HAS3
RXFP1	1.56	0.32	2.36	0.0033	0.0737	RXFP1
AFAP1	6.97	5.73	2.36	0.0222	0.1794	AFAP1
VPS36	4.99	3.76	2.36	0.0262	0.1954	VPS36
ZDHHC14	4.12	2.87	2.37	0.0011	0.0476	ZDHHC14
SAMHD1	7.02	5.78	2.37	0.0034	0.0739	SAMHD1
CEBPD	3.6	2.36	2.37	0.0073	0.1058	CEBPD
STARD4	5.55	4.31	2.37	0.0075	0.1074	STARD4
ZC3H12A	5.1	3.85	2.38	0.0041	0.0805	ZC3H12A
HSPA6	4.68	3.43	2.38	0.0215	0.1775	HSPA6
C5orf15	7.18	5.91	2.4	0.0012	0.0492	C5orf15
MAPK8IP2	2.51	1.24	2.4	0.0041	0.0801	MAPK8IP2
MAP3K13	4.45	3.18	2.41	0.0008	0.0403	MAP3K13
NFKBIA	7.93	6.66	2.42	0.0017	0.0561	NFKBIA

IFIT1B	1.28	0	2.42	0.0022	0.0634	IFIT1B
SCN3A	1.48	0.2	2.42	0.0075	0.1074	SCN3A
GBP6	1.4	0.12	2.43	0.0089	0.1162	GBP6
FBXO6	4.69	3.4	2.44	0.0007	0.0383	FBXO6
STK10	5.48	4.19	2.44	0.0097	0.1212	STK10
VEGFC	7.88	6.59	2.45	0.0002	0.0269	VEGFC
GRB10	8.68	7.39	2.45	0.0006	0.0366	GRB10
SLC25A28	4.7	3.41	2.45	0.0033	0.0739	SLC25A28
BTN3A2	6.67	5.38	2.45	0.0059	0.0961	BTN3A2
CTSO	3.59	2.29	2.46	0.007	0.1028	CTSO
WHAMMP3	3.3	2	2.46	0.0097	0.1218	WHAMMP3
CYP2J2	3.15	1.85	2.47	0.003	0.0711	CYP2J2
IGFBP3	9.84	8.53	2.48	0.0034	0.0739	IGFBP3
CHIC1	3.84	2.52	2.49	2.75E-05	0.0154	CHIC1
PLAUR	8.14	6.82	2.5	0.0003	0.0289	PLAUR
ZDHHC9	6.23	4.91	2.5	0.0016	0.0552	ZDHHC9
TMEM41B	5.96	4.63	2.5	0.005	0.0879	TMEM41B
DNAJC18	3.75	2.43	2.5	0.0225	0.1807	DNAJC18
TBC1D7	4.97	3.64	2.51	9.51E-05	0.0225	TBC1D7
DBT	4.79	3.46	2.51	0.0019	0.0592	DBT
ITGB8	5.66	4.33	2.52	0.0005	0.0346	ITGB8
PSORS1C1	3.73	2.4	2.52	0.0009	0.0428	PSORS1C1
FMNL2	5.58	4.25	2.52	0.0056	0.094	FMNL2
STEAP4	4.5	3.16	2.53	0.0003	0.0293	STEAP4
IL2RG	1.46	0.12	2.53	0.0179	0.1605	IL2RG
OLR1	1.94	0.6	2.54	0.0407	0.2456	OLR1
FOXD1	5.36	4.01	2.55	0.0002	0.0269	FOXD1
RP2	6.31	4.96	2.55	0.0016	0.0561	RP2
CACNA1I	1.59	0.24	2.56	0.0005	0.0364	CACNA1I
KIAA1033	4.4	3.04	2.56	0.0017	0.0561	KIAA1033
JAZF1	5.08	3.73	2.56	0.0024	0.0649	JAZF1
MICB	6.62	5.26	2.56	0.0151	0.1493	MICB
ZCCHC16	2.88	1.52	2.57	3.46E-05	0.0157	ZCCHC16
NCSTN	7.9	6.54	2.57	0.0007	0.039	NCSTN
ZNF711	4.28	2.92	2.57	0.0015	0.054	ZNF711
TCF4	4.38	3.02	2.58	0.0017	0.0572	TCF4
FGF5	4.7	3.33	2.58	0.0098	0.1219	FGF5
RAP1B	7.58	6.21	2.59	0.0046	0.0848	RAP1B
NAPA	8.42	7.04	2.6	0.0005	0.0342	NAPA
SP140L	7.56	6.18	2.6	0.0039	0.0792	SP140L
CPED1	2.66	1.28	2.6	0.0166	0.1554	CPED1
SEMA3F	6.57	5.19	2.61	9.25E-05	0.0223	SEMA3F
GABRA6	1.38	0	2.61	0.0006	0.0366	GABRA6
IL8	10.53	9.14	2.61	0.0011	0.0469	IL8
ABCD1	6.96	5.58	2.61	0.0026	0.0672	ABCD1
SGCE	4.29	2.91	2.61	0.0028	0.0693	SGCE
TXNIP	7.43	6.04	2.61	0.0079	0.1102	TXNIP

TRIM14	7.3	5.91	2.62	0.0009	0.0416	TRIM14
AMOTL2	6.3	4.92	2.62	0.0025	0.0666	AMOTL2
MAPRE1	7.91	6.52	2.63	0.0016	0.0558	MAPRE1
EPHA7	4.74	3.34	2.63	0.002	0.0602	EPHA7
PLOD2	9.13	7.72	2.65	0.0153	0.1502	PLOD2
RNF7	6.16	4.75	2.66	0.0009	0.0422	RNF7
WIPF1	4.05	2.64	2.66	0.0016	0.0559	WIPF1
CLEC2B	4.11	2.69	2.67	0.0183	0.1623	CLEC2B
ICAM1	6.86	5.44	2.69	0.0003	0.0293	ICAM1
NID2	3.09	1.67	2.69	0.0068	0.1018	NID2
TTC32	3.91	2.48	2.7	0.0006	0.0366	TTC32
TCN2	3.66	2.23	2.7	0.0129	0.1393	TCN2
PGAM1	9.1	7.66	2.71	0.0009	0.0422	PGAM1
KLF6	7.75	6.32	2.71	0.0039	0.0792	KLF6
JAK2	4.85	3.41	2.72	0.0024	0.0654	JAK2
DYNLT3	6.66	5.21	2.73	0.0133	0.1412	DYNLT3
RAET1L	2.88	1.42	2.74	0.0079	0.11	RAET1L
HIPK3	6.16	4.7	2.76	0.0003	0.0293	HIPK3
GPR180	5.29	3.83	2.76	0.0026	0.0674	GPR180
FBXO32	5.41	3.94	2.77	3.35E-05	0.0157	FBXO32
PLA1A	2.25	0.78	2.77	0.0023	0.0636	PLA1A
CKAP4	7.44	5.97	2.77	0.0028	0.0701	CKAP4
FYB	3.61	2.14	2.77	0.0061	0.0973	FYB
RND1	3.01	1.54	2.77	0.0145	0.1465	RND1
LDLR	9.67	8.2	2.77	0.0166	0.1554	LDLR
ERO1L	8.76	7.28	2.78	4.72E-05	0.0176	ERO1L
SERPING1	1.6	0.13	2.78	7.30E-05	0.0214	SERPING1
GNB4	4.56	3.09	2.78	0.0002	0.0257	GNB4
CD274	4.71	3.24	2.78	0.0003	0.0293	CD274
GKN2	2.76	1.28	2.78	0.0067	0.1017	GKN2
CHSY3	3.05	1.58	2.78	0.0171	0.1577	CHSY3
ANXA3	7.64	6.16	2.79	0.0005	0.036	ANXA3
HHLA3	4.48	2.99	2.8	0.0003	0.0293	HHLA3
RBCK1	6.64	5.15	2.8	0.0006	0.0366	RBCK1
IL22RA1	3.43	1.94	2.8	0.0022	0.0634	IL22RA1
IFI44L	6.92	5.44	2.8	0.0074	0.1066	IFI44L
N4BP1	6.3	4.81	2.81	8.96E-06	0.0117	N4BP1
LGMN	7.39	5.9	2.81	0.0013	0.0522	LGMN
GRAMD1B	7.9	6.4	2.82	0.0002	0.0276	GRAMD1B
TAP2	8.06	6.57	2.82	0.0014	0.0535	TAP2
EPHA4	2.81	1.32	2.82	0.0023	0.0636	EPHA4
KCTD5	7.25	5.75	2.83	0.0002	0.0269	KCTD5
UNC93B1	6.87	5.36	2.86	0.0004	0.0319	UNC93B1
CCNJ	4.26	2.74	2.86	0.0008	0.0408	CCNJ
TMEM62	6.37	4.85	2.87	0.0008	0.0403	TMEM62
RAB21	6.29	4.76	2.88	0.0005	0.0352	RAB21
SERPINE1	9.63	8.1	2.89	0.004	0.0801	SERPINE1

PNP	6.37	4.83	2.9	1.98E-05	0.0142	PNP
KLHDC7B	1.63	0.1	2.9	0.012	0.1349	KLHDC7B
SH3BGRL	6.43	4.88	2.93	0.0012	0.0492	SH3BGRL
C3AR1	3.02	1.47	2.93	0.0166	0.1555	C3AR1
HES4	5.41	3.86	2.94	0.0003	0.0293	HES4
IL6	6.27	4.71	2.94	0.0084	0.1133	IL6
PION	4.37	2.81	2.95	5.15E-05	0.0176	PION
NR4A1	3.64	2.07	2.96	0.0167	0.1558	NR4A1
RBM43	4.34	2.76	2.98	0.0149	0.1481	RBM43
C21orf91	6.45	4.87	2.99	0.0002	0.0269	C21orf91
RDX	7.16	5.58	3	0.0004	0.0309	RDX
MFSD12	3.52	1.94	3	0.0071	0.1034	MFSD12
GAL3ST4	3.08	1.49	3	0.0072	0.1054	GAL3ST4
TLR4	3.31	1.72	3	0.0248	0.1908	TLR4
TMEM30A	6.58	4.99	3.01	4.00E-05	0.0176	TMEM30A
SP100	6.63	5.04	3.01	0.0004	0.0336	SP100
TMEM209	5.9	4.3	3.02	0.0008	0.0395	TMEM209
RECK	3.18	1.58	3.02	0.0093	0.1186	RECK
IL7	2.54	0.94	3.02	0.0102	0.1245	IL7
PRR15	2.24	0.63	3.04	0.0067	0.1012	PRR15
BIRC3	5.91	4.31	3.04	0.0088	0.1156	BIRC3
FLT3LG	3.09	1.49	3.05	0.0007	0.039	FLT3LG
LYPD1	4.21	2.59	3.06	2.27E-05	0.0154	LYPD1
TRIM38	6.83	5.21	3.08	0.0004	0.0308	TRIM38
CASP10	3.68	2.06	3.08	0.0109	0.1284	CASP10
HEXDC	2.75	1.12	3.09	0.0043	0.0818	HEXDC
OGFR	6.54	4.9	3.1	0.0005	0.0342	OGFR
NFE2L3	6.9	5.26	3.11	1.51E-05	0.0126	NFE2L3
BCL2A1	4.86	3.22	3.12	0.0105	0.1263	BCL2A1
SEMA3D	4.29	2.65	3.12	0.0131	0.1406	SEMA3D
PLAT	8.38	6.73	3.13	0.0008	0.0396	PLAT
PNRC2	7.95	6.3	3.15	5.33E-05	0.0179	PNRC2
ST6GALNAC2	3.03	1.38	3.15	0.0025	0.0667	ST6GALNAC2
ENDOD1	4.53	2.88	3.16	0.0011	0.0465	ENDOD1
PML	5.29	3.62	3.16	0.015	0.1485	PML
SLITRK2	1.79	0.12	3.18	0.0001	0.0253	SLITRK2
STAT2	8.02	6.36	3.18	0.0005	0.0342	STAT2
MUC1	2.4	0.73	3.18	0.0007	0.0386	MUC1
LYPLA1	5.37	3.7	3.19	0.0001	0.0253	LYPLA1
EFR3A	6.57	4.89	3.19	0.0002	0.0269	EFR3A
SCARB2	7.12	5.45	3.19	0.0006	0.0366	SCARB2
EOGT	3.4	1.72	3.2	0.0142	0.1446	EOGT
SERPINB2	4.45	2.77	3.22	0.0075	0.1074	SERPINB2
GPD1L	4.46	2.76	3.25	0.0003	0.0299	GPD1L
CSGALNACT2	5.72	4.01	3.26	0.0056	0.0939	CSGALNACT2
HIST3H2A	6.34	4.63	3.27	0.0004	0.0312	HIST3H2A
PCGF5	4.79	3.08	3.28	0.0022	0.0628	PCGF5

TAPBPL	1.99	0.28	3.28	0.0063	0.0987	TAPBPL
ENOPH1	6.19	4.47	3.29	7.01E-05	0.0214	ENOPH1
HTATIP2	4.52	2.8	3.29	0.0023	0.0636	HTATIP2
ZNFX1	8.05	6.33	3.29	0.0024	0.0653	ZNFX1
CXCL5	4.84	3.12	3.29	0.0077	0.1088	CXCL5
COL13A1	6.92	5.2	3.3	0.0012	0.0499	COL13A1
ZEB1	5.68	3.96	3.31	0.0008	0.0395	ZEB1
LRCH2	3.87	2.13	3.33	0.0001	0.0253	LRCH2
LATS2	3.84	2.1	3.34	0.0009	0.0434	LATS2
ODF3B	2.76	1.01	3.37	0.0005	0.0352	ODF3B
TMEM132A	5.35	3.6	3.37	0.002	0.0595	TMEM132A
OAS3	9.43	7.68	3.37	0.0063	0.0986	OAS3
IRF1	5.49	3.74	3.37	0.0222	0.1794	IRF1
NFKBIZ	7.19	5.44	3.38	0.0038	0.0786	NFKBIZ
TNFAIP3	6.79	5.02	3.42	0.0029	0.0704	TNFAIP3
KRT6B	2.74	0.94	3.46	0.0022	0.0634	KRT6B
RUNX2	4.46	2.66	3.47	0.0013	0.0505	RUNX2
GBP3	6.77	4.95	3.53	0.0002	0.0263	GBP3
HLA-F	2.2	0.38	3.53	0.0008	0.0407	HLA-F
CHRNA1	3.23	1.39	3.59	0.0011	0.0468	CHRNA1
GBP1P1	1.85	0	3.6	0.0046	0.0851	GBP1P1
TRIM5	7.64	5.79	3.61	0.0001	0.0235	TRIM5
ARL6IP1	7.43	5.58	3.61	0.0006	0.0366	ARL6IP1
IFIT5	6.08	4.22	3.61	0.0014	0.0535	IFIT5
SLC2A12	3.95	2.09	3.61	0.0038	0.0787	SLC2A12
IL12A	4.24	2.39	3.62	0.0003	0.0293	IL12A
CARD16	2.2	0.34	3.62	0.0139	0.1432	CARD16
RICTOR	7.47	5.61	3.63	0.0004	0.0311	RICTOR
AIM2	1.98	0.12	3.64	0.0041	0.0804	AIM2
C15orf48	5.77	3.9	3.64	0.0069	0.1026	C15orf48
IL1RAP	4.86	2.99	3.66	0.001	0.0458	IL1RAP
CXCL1	8.54	6.65	3.69	0.0078	0.1097	CXCL1
LY6E	8.32	6.43	3.7	0.0002	0.0278	LY6E
HOXD8	2.84	0.94	3.72	0.0013	0.0523	HOXD8
C10orf57	6.7	4.79	3.74	4.12E-06	0.0095	C10orf57
RAB42	3.87	1.96	3.76	0.0089	0.1159	RAB42
SLC44A1	6.06	4.13	3.8	1.37E-06	0.0078	SLC44A1
LIX1L	6.68	4.76	3.8	0.0027	0.0677	LIX1L
ACSL5	2.67	0.74	3.8	0.0034	0.0739	ACSL5
FUBP3	6.78	4.84	3.85	0.0017	0.0561	FUBP3
CCL20	3.09	1.15	3.86	0.0252	0.1918	CCL20
ID3	3.95	1.99	3.89	0.001	0.0452	ID3
IL28A	1.96	0	3.9	0.0027	0.0683	IL28A
PCDH17	2.11	0.13	3.95	0.0002	0.0269	PCDH17
TDRD7	5.54	3.56	3.95	0.0002	0.0284	TDRD7
TNFAIP6	3.68	1.7	3.95	0.0029	0.0704	TNFAIP6
IL7R	7.95	5.97	3.96	0.0029	0.0706	IL7R

NMI	6.13	4.14	3.97	4.67E-05	0.0176	NMI
LRRC38	5.68	3.67	4.04	0.0026	0.0673	LRRC38
HLA-B	2.64	0.62	4.06	0.0005	0.0362	HLA-B
PPM1K	5.52	3.48	4.09	0.0003	0.0293	PPM1K
SPINK6	2.38	0.35	4.09	0.0061	0.0971	SPINK6
FNDC3A	7.3	5.26	4.1	0.0006	0.0366	FNDC3A
ARL14	5.68	3.65	4.1	0.0016	0.0552	ARL14
B2M	11.51	9.46	4.13	1.09E-05	0.0121	B2M
SIDT1	6.02	3.97	4.14	0.0059	0.0961	SIDT1
PNPT1	8.39	6.34	4.15	4.53E-05	0.0176	PNPT1
IL11	3.5	1.45	4.15	0.0029	0.0704	IL11
PAPL	4.73	2.68	4.16	0.0034	0.0739	PAPL
SC5DL	3.95	1.9	4.17	0.0011	0.0468	SC5DL
HINT3	4.7	2.64	4.18	0.0015	0.0537	HINT3
PARP12	6.28	4.2	4.22	0.0001	0.0235	PARP12
NT5E	7.55	5.46	4.24	0.0001	0.0253	NT5E
GIMAP2	2.5	0.4	4.28	0.0002	0.0278	GIMAP2
IL15RA	5.41	3.31	4.28	0.0004	0.0329	IL15RA
ZC3HAV1	9.42	7.3	4.32	0.0012	0.0503	ZC3HAV1
IGFBP6	10.12	8.01	4.33	0.0004	0.0319	IGFBP6
PDCD1LG2	5.57	3.45	4.35	0.0029	0.0704	PDCD1LG2
CCR1	2.13	0	4.36	0.0003	0.0294	CCR1
ID1	9.09	6.97	4.36	0.0239	0.1873	ID1
STAT1	10.82	8.69	4.38	0.0008	0.04	STAT1
PMAIP1	8.44	6.28	4.46	0.0002	0.0276	PMAIP1
SGPP1	6.87	4.71	4.48	0.0001	0.0253	SGPP1
FAM46A	7.27	5.1	4.49	0.0001	0.0253	FAM46A
CD68	9.3	7.12	4.51	0.0001	0.0253	CD68
PLSCR1	9.28	7.1	4.52	0.0012	0.0492	PLSCR1
ST8SIA4	3.1	0.91	4.58	0.0007	0.039	ST8SIA4
BTC	5.65	3.43	4.64	0.0002	0.0278	BTC
S100P	4.71	2.49	4.65	0.0018	0.0573	S100P
TFPI2	10.2	7.98	4.66	1.87E-06	0.0078	TFPI2
DTX3L	8.24	6.01	4.7	0.0002	0.0269	DTX3L
GBP2	6.1	3.86	4.73	3.08E-05	0.0154	GBP2
PSAT1	5.89	3.63	4.78	0.0007	0.038	PSAT1
C4orf33	4.35	2.1	4.79	0.0001	0.0253	C4orf33
C19orf66	6.71	4.43	4.85	8.92E-05	0.0222	C19orf66
PHF11	4.92	2.62	4.93	0.0003	0.0293	PHF11
CD55	5.87	3.57	4.93	0.0005	0.0351	CD55
IRF7	8.06	5.75	4.94	0.0013	0.0511	IRF7
IFNB1	2.79	0.46	5	3.27E-05	0.0157	IFNB1
DDX60	7.21	4.88	5.02	0.0029	0.0704	DDX60
PARP14	7.83	5.49	5.05	0.0009	0.0434	PARP14
DDX60L	7.46	5.04	5.35	0.0002	0.0269	DDX60L
LAMP3	7.48	5.06	5.35	0.0005	0.0363	LAMP3
C1S	5.34	2.89	5.45	0.0003	0.0299	C1S

HELZ2	7.39	4.94	5.48	0.0006	0.0373	HELZ2
C1R	6.87	4.41	5.52	0.0003	0.0293	C1R
ZMYM6NB	5.98	3.48	5.63	0.0002	0.0269	ZMYM6NB
CSF3	3.04	0.55	5.64	0.0004	0.0311	CSF3
APOBEC3G	3.73	1.23	5.64	0.003	0.0712	APOBEC3G
PLSCR4	5.5	3	5.67	0.0002	0.0278	PLSCR4
TRIM21	6.62	4.09	5.79	0.0002	0.0275	TRIM21
SAMD9	7.67	5.13	5.81	7.13E-05	0.0214	SAMD9
CSF1	6.66	4.11	5.86	0.0012	0.05	CSF1
CBX1	4.38	1.82	5.88	0.0005	0.0352	CBX1
CFH	4.26	1.69	5.92	0.0003	0.03	CFH
KRT75	4.78	2.2	6	0.0014	0.0535	KRT75
CTSS	2.94	0.34	6.05	0.0079	0.1098	CTSS
KRT17	4.15	1.55	6.08	0.003	0.0712	KRT17
IL29	2.74	0.12	6.17	0.0002	0.0257	IL29
IFI16	7.4	4.74	6.32	0.0007	0.0391	IFI16
SECTM1	7.82	5.16	6.34	0.0002	0.0254	SECTM1
THEMIS2	5.35	2.62	6.64	0.0003	0.0297	THEMIS2
APOL6	7.55	4.81	6.7	1.18E-05	0.0121	APOL6
PTGS2	6.96	4.21	6.7	0.0005	0.035	PTGS2
PLEKHA4	3.15	0.38	6.83	0.0014	0.0535	PLEKHA4
TMEM27	7.16	4.38	6.87	1.52E-08	0.0003	TMEM27
TLR3	3.99	1.2	6.91	1.81E-05	0.0135	TLR3
DDX58	9.27	6.43	7.15	0.0007	0.038	DDX58
TMEM229B	4.17	1.33	7.18	0.0003	0.0293	TMEM229B
ACE2	3.45	0.6	7.21	0.0015	0.0547	ACE2
HSH2D	6.11	3.25	7.24	7.61E-05	0.0219	HSH2D
TYMP	6.89	4.01	7.34	0.0017	0.057	TYMP
PARP9	6.32	3.44	7.35	0.0003	0.0299	PARP9
ATP10A	4.3	1.41	7.39	0.0019	0.0592	ATP10A
APOL3	3.75	0.86	7.41	0.0004	0.0342	APOL3
HLA-C	6.03	3.14	7.41	0.001	0.0457	HLA-C
IFI44	9.5	6.58	7.55	0.0004	0.0319	IFI44
NEK7	5.99	3.06	7.63	4.99E-05	0.0176	NEK7
TMEM140	6.72	3.78	7.66	4.63E-05	0.0176	TMEM140
PARP10	6.43	3.47	7.77	2.89E-05	0.0154	PARP10
USP18	7.49	4.51	7.89	2.84E-05	0.0154	USP18
IFI35	8.46	5.43	8.17	5.13E-05	0.0176	IFI35
SP110	4.57	1.51	8.34	0.0001	0.0235	SP110
EPSTI1	4.02	0.91	8.61	0.0003	0.0293	EPSTI1
TRANK1	6.39	3.24	8.88	0.0007	0.0386	TRANK1
GMPR	5.24	2.08	8.92	0.0003	0.0297	GMPR
HRASLS2	3.57	0.41	8.92	0.0011	0.0469	HRASLS2
IFITM2	9.56	6.37	9.15	0.0001	0.0253	IFITM2
UBE2L6	8.99	5.73	9.56	5.99E-06	0.0104	UBE2L6
HERC6	6.84	3.57	9.65	0.0016	0.0559	HERC6
CEACAM1	4.13	0.83	9.87	9.33E-05	0.0223	CEACAM1

TAP1	9.02	5.68	10.13	6.26E-05	0.0203	TAP1
LGALS9	3.4	0	10.55	0.0002	0.0271	LGALS9
IFIH1	7.89	4.47	10.72	6.67E-06	0.0104	IFIH1
CASP1	3.58	0.12	10.98	0.0005	0.0363	CASP1
MMP13	7.41	3.94	11.08	0.0003	0.0293	MMP13
CXCL10	3.94	0.45	11.19	0.0023	0.0645	CXCL10
LGALS3BP	10.02	6.53	11.23	0.0003	0.0293	LGALS3BP
CFB	4.59	1.08	11.37	0.0001	0.0253	CFB
MUC13	5.15	1.63	11.43	0.0002	0.028	MUC13
SAMD9L	7.12	3.59	11.49	7.98E-05	0.0219	SAMD9L
OAS1	9.8	6.26	11.62	0.0018	0.0573	OAS1
NCF2	5.11	1.55	11.76	0.0003	0.03	NCF2
IL4I1	4.39	0.75	12.48	0.0018	0.0573	IL4I1
CXCL11	4.59	0.93	12.69	0.0012	0.0496	CXCL11
IFITM3	10.92	7.25	12.77	0.0001	0.0253	IFITM3
HERC5	7.34	3.66	12.84	0.0003	0.0299	HERC5
PSMB9	6.74	3.02	13.14	6.13E-06	0.0104	PSMB9
IFITM1	10.97	7.24	13.34	0.0001	0.0253	IFITM1
NLRC5	6.02	2.26	13.59	8.01E-05	0.0219	NLRC5
ISG20	7.17	3.35	14.16	0.0009	0.043	ISG20
SLC15A3	3.97	0.12	14.39	6.10E-05	0.0202	SLC15A3
OAS2	8.66	4.76	14.92	0.0021	0.0615	OAS2
ISG15	12.22	8.26	15.54	0.0004	0.0342	ISG15
UBA7	5.02	1.03	15.99	8.92E-05	0.0222	UBA7
IDO1	6.51	2.5	16.14	0.0002	0.0269	IDO1
IFIT2	11.08	6.95	17.55	4.45E-05	0.0176	IFIT2
IFIT1	10.96	6.81	17.84	0.0003	0.0286	IFIT1
GBP1	7.96	3.66	19.67	8.52E-05	0.0222	GBP1
MX1	11.03	6.73	19.79	0.0005	0.0363	MX1
APOL1	5.23	0.91	19.97	5.16E-05	0.0176	APOL1
RARRES3	6.71	2.36	20.33	0.0059	0.0961	RARRES3
IFI6	12.33	7.83	22.6	0.0007	0.0381	IFI6
CCL5	5.11	0.53	23.84	0.0003	0.03	CCL5
TNFSF10	5.52	0.93	23.97	0.0002	0.0278	TNFSF10
RTP4	4.95	0.23	26.29	0.0005	0.0352	RTP4
GBP5	5.45	0.71	26.8	0.0002	0.0257	GBP5
GBP4	5.94	0.81	35	1.44E-05	0.0126	GBP4
IFIT3	7.72	2.45	38.62	0.0002	0.0263	IFIT3
XAF1	5.41	0	42.59	1.08E-06	0.0078	XAF1
OASL	9.28	3.73	46.86	0.0002	0.0257	OASL
MX2	7.69	1.96	52.94	0.0001	0.0238	MX2
CMPK2	8.01	2.08	60.85	0.0002	0.0284	CMPK2
RSAD2	9.1	3.11	63.44	0.0003	0.0293	RSAD2
IFI27	8.06	2	66.41	0.0003	0.0293	IFI27
TRIM22	6.52	0.46	66.79	1.22E-05	0.0121	TRIM22
BST2	10.24	1.92	320.01	0.0002	0.0278	BST2
