# Supplementary material for: The evolutionarily conserved long non‐coding RNA LINC00261 drives neuroendocrine prostate cancer proliferation and metastasis via distinct nuclear and cytoplasmic mechanisms
Source: Mol Oncol. 2021 Apr 26;15(7):1921–41. doi: 10.1002/1878-0261.12954 (PMC8253100; doi:10.1002/1878-0261.12954)
Supplement: Supplementary file 10 — Table S4. Common genes between the predicted miR‐8485 targets and genes down‐regulated upon LINC00261 silencing in PC‐3 cells. For each of the 77 genes, fold change and associated P‐value upon LINC00261 silencing are shown, along with identification of the miRNA‐binding tools that predict targeting by miR‐8485. [file MOL2-15-1921-s007.pdf]

Supplementary Table 4: Common genes between the predicted miR-8485 targets and genes down-regulated upon *LINC00261* silencing in PC-3 cells

Genes	siRNA Avg (log2)	NC Avg (log2)	Fold Change	P-val	FDR P-val	TargetScan	MirDB	miRWALK	RNA22	Total
TOR1B	2.97	5.18	-4.63	6.00E-04	3.66E-02	1	1	1	1	4
PADI2	0.1	1.82	-3.28	5.90E-03	9.61E-02	1	1	1	1	4
SLC4A7	5.78	7.48	-3.24	3.80E-03	7.85E-02	1	1	1	1	4
MBNL3	1.56	3.01	-2.73	3.80E-03	7.86E-02	1	1	1	1	4
ESM1	3.98	5.2	-2.33	2.05E-02	1.73E-01	1	1	1	1	4
KIF5C	1.74	2.97	-2.33	6.00E-04	3.66E-02	1	1	1	1	4
PRKCA	5.82	7.01	-2.29	8.00E-03	1.11E-01	1	1	1	1	4
CBX2	4.07	5.26	-2.28	1.40E-02	1.44E-01	1	1	1	1	4
MMAB	4.29	5.44	-2.22	1.38E-02	1.43E-01	1	1	1	1	4
IGSF9	2.45	3.46	-2.02	2.60E-03	6.76E-02	1	1	1	1	4
ERLIN2	3.92	5.22	-2.47	4.30E-03	8.19E-02	1	1	1	0	3
ANKRD22	2.07	3.37	-2.46	1.32E-02	1.41E-01	1	0	1	1	3
RAB31	3.5	4.78	-2.43	3.60E-03	7.73E-02	1	0	1	1	3
RAB11FIP4	1.3	2.43	-2.18	2.10E-02	1.75E-01	1	1	1	0	3
SETD7	5.57	6.64	-2.1	2.20E-03	6.28E-02	1	1	1	0	3
MAD2L1	5.03	6.78	-3.38	1.70E-03	5.71E-02	1	1	0	0	2
MYH15	1.89	3.38	-2.8	2.37E-02	1.86E-01	1	0	1	0	2
BRMS1L	3.49	4.92	-2.7	3.10E-03	7.18E-02	1	1	0	0	2
HMGA2	6.17	7.57	-2.66	7.00E-04	3.90E-02	1	0	0	1	2
TTC3	5.66	7.05	-2.61	2.04E-02	1.72E-01	1	1	0	0	2
CYFIP2	2.79	4.17	-2.59	1.20E-03	5.00E-02	1	1	0	0	2
SAMD5	1.61	2.97	-2.56	3.50E-02	2.25E-01	0	0	1	1	2
DPYSL3	4.32	5.65	-2.52	6.00E-04	3.73E-02	1	0	1	0	2
BZW1	8.55	9.86	-2.48	8.66E-05	2.22E-02	1	0	0	1	2
VCAN	2.69	3.98	-2.46	4.71E-02	2.65E-01	1	0	0	1	2
AIF1L	3	4.29	-2.45	1.55E-02	1.51E-01	1	0	1	0	2
ALDH1A3	6.83	8.08	-2.38	3.20E-03	7.28E-02	1	1	0	0	2
SLC26A2	4.21	5.45	-2.37	1.07E-02	1.27E-01	1	1	0	0	2
SCD	6.92	8.14	-2.33	2.62E-02	1.95E-01	1	0	1	0	2
PDE5A	3.81	5.03	-2.32	1.00E-04	2.53E-02	1	1	0	0	2
RDH10	6.26	7.4	-2.21	9.60E-03	1.21E-01	1	1	0	0	2
MLEC	6.83	7.97	-2.2	6.00E-04	3.66E-02	1	1	0	0	2
PTGFRN	3.38	4.52	-2.2	1.40E-02	1.44E-01	1	1	0	0	2
DENR	6.17	7.29	-2.18	6.00E-04	3.66E-02	1	1	0	0	2
SAPCD2	3.41	4.52	-2.17	3.30E-03	7.39E-02	1	0	1	0	2
BSPRY	2.24	3.29	-2.07	3.51E-02	2.26E-01	1	0	1	0	2
CACYBP	5.42	6.45	-2.05	7.20E-03	1.05E-01	1	1	0	0	2
DESI2	4.78	5.78	-2	1.08E-02	1.28E-01	1	1	0	0	2
AXL	4.81	6.59	-3.43	2.00E-04	2.78E-02	0	0	1	0	1
FABP4	1.64	3.39	-3.37	2.90E-03	7.06E-02	1	0	0	0	1
ELOVL5	6.62	8.24	-3.08	1.52E-05	1.26E-02	1	0	0	0	1
PRKDC	6.68	8.28	-3.02	1.59E-05	1.26E-02	1	0	0	0	1
CALB2	0.48	1.95	-2.78	1.89E-02	1.65E-01	1	0	0	0	1
TULP4	2.75	4.2	-2.73	2.88E-02	2.04E-01	0	0	1	0	1
CROT	3.56	5	-2.72	6.87E-05	2.14E-02	0	0	1	0	1
SHH	1.58	2.96	-2.61	4.50E-03	8.39E-02	1	0	0	0	1
MAP7D2	1.41	2.77	-2.56	1.00E-04	2.53E-02	0	0	1	0	1
POLR1A	5.47	6.82	-2.55	1.93E-02	1.67E-01	0	0	0	1	1
MT1F	3.43	4.73	-2.47	3.80E-03	7.86E-02	1	0	0	0	1

**ABCA13**	1.84	3.13	-2.45	3.90E-03	7.92E-02	1	0	0	0	1
**SFT2D2**	5.27	6.56	-2.45	5.50E-03	9.27E-02	1	0	0	0	1
**MYH9**	8.38	9.67	-2.44	1.10E-03	4.76E-02	1	0	0	0	1
**PPARA**	3.4	4.66	-2.4	4.30E-03	8.25E-02	1	0	0	0	1
**PRR11**	5.93	7.18	-2.37	6.00E-04	3.66E-02	0	0	1	0	1
**CIT**	4.65	5.84	-2.28	8.80E-03	1.16E-01	1	0	0	0	1
**SARDH**	1.92	3.08	-2.23	2.77E-02	2.00E-01	1	0	0	0	1
**RCOR2**	1.28	2.42	-2.2	4.00E-04	3.11E-02	1	0	0	0	1
**CRISPLD2**	1.1	2.23	-2.19	1.29E-02	1.40E-01	1	0	0	0	1
**SYTL1**	1.82	2.95	-2.18	1.19E-02	1.35E-01	0	0	0	1	1
**HIST1H4E**	6.36	7.46	-2.14	1.37E-02	1.43E-01	1	0	0	0	1
**MPND**	1.21	2.3	-2.13	4.66E-02	2.64E-01	1	0	0	0	1
**PALM3**	2.43	3.5	-2.11	3.03E-02	2.08E-01	0	0	1	0	1
**CTH**	2.98	4.05	-2.09	8.00E-04	4.12E-02	1	0	0	0	1
**PTPN14**	4.89	5.95	-2.09	6.00E-04	3.77E-02	1	0	0	0	1
**AKAP12**	7.25	8.31	-2.08	1.04E-02	1.26E-01	1	0	0	0	1
**RRAS2**	4.25	5.31	-2.08	7.00E-04	3.88E-02	1	0	0	0	1
**GATSL2**	1.44	2.49	-2.07	1.77E-02	1.60E-01	1	0	0	0	1
**POLA1**	4.71	5.76	-2.07	9.50E-03	1.20E-01	1	0	0	0	1
**EIF4B**	7.57	8.6	-2.05	2.69E-02	1.98E-01	1	0	0	0	1
**HNRNPA0**	4.25	5.28	-2.04	3.00E-04	2.93E-02	0	0	1	0	1
**PTPLB**	6.18	7.21	-2.04	1.80E-03	5.83E-02	1	0	0	0	1
**KIAA1462**	5.19	6.21	-2.03	2.70E-03	6.83E-02	1	0	0	0	1
**CCNB1IP1**	5.3	6.31	-2.02	8.00E-04	4.12E-02	1	0	0	0	1
**REPS2**	2.71	3.72	-2.02	1.70E-03	5.61E-02	1	0	0	0	1
**COMMD8**	3.65	4.66	-2.01	8.40E-03	1.14E-01	1	0	0	0	1
**DFFA**	5.71	6.71	-2.01	1.06E-02	1.27E-01	1	0	0	0	1
**RALGAPA2**	4.18	5.18	-2	1.72E-02	1.58E-01	0	0	1	0	1
